# Supplementary material for: Resolving the cause of recurrent Plasmodium vivax malaria probabilistically
Source: Nat Commun. 2019 Dec 6;10:5595. doi: 10.1038/s41467-019-13412-x (PMC6898227; doi:10.1038/s41467-019-13412-x)
Supplement: Supplementary file 1 — Supplementary Information [file 41467_2019_13412_MOESM1_ESM.pdf]

Resolving the cause of recurrent *Plasmodium vivax* malaria probabilistically

Taylor and Watson *et al.*

Supplementary Tables

Parameter	Meaning	Logit transform†	Prior	Posterior median [95% CI]
λ_{VHX}	Reinfection rate in the VHX study		$\mathcal{G}(50, 6 \cdot 10^4)$	0.00095 [0.00077;0.00115]
δ	Reinfection rate decrease factor (VHX to BPD)		$\mathcal{N}(0.66, 0.15)$	0.47 [0.36;0.63]
γ	Late relapse rate		$\mathcal{G}(50, 2.5 \cdot 10^3)$	0.0086 [0.0075;0.0099]
λ_{RC}	Recrudescence rate		$\mathcal{G}(100, 10^3)$	0.095 [0.077;0.115]
q	Mixture weight: early (periodic) versus late (constant-rate) relapse	yes	$\mathcal{N}(0, 0.25)$	0.45 [0.29;0.61]
μ_{AS}	Scale parameter for early relapse (AS)		$\mathcal{N}(27, 1)$	29 [28;30]
μ_{CQ}	Scale parameter for early relapse (CQ/PMQ+)		$\mathcal{N}(45, 1)$	46 [45;47]
k_{AS}	Shape parameter for early relapse (AS)		$\mathcal{N}(5, 0.5)$	4.1 [3.8;4.5]
k_{CQ}	Shape parameter for early relapse (CQ/PMQ+)		$\mathcal{N}(5, 0.5)$	5.2 [4.8;5.7]
p_n^{AS}, p_n^{CQ}	Individual mixture weight: reinfection versus relapse or recrudescence (no PMQ)	yes	$\mathcal{N}(p^{\mu}_{\mathrm{AS/CQ}}, p^{\sigma}_{\mathrm{AS/CQ}})$	-2.3 [-9.8;5.2]*
$p_n^{\mathrm{PMQ+}}$	Individual mixture weight: reinfection versus relapse or recrudescence (PMQ+)	yes	$\mathcal{N}(p^{\mu}_{\mathrm{PMQ+}}, p^{\sigma}_{\mathrm{PMQ+}}))$	3.5 [2.8;4.2]*
$p^{\mu}_{\mathrm{AS/CQ}}$	Population mean reinfection mixture weight (no PMQ)		$\mathcal{N}(\mathrm{logit}(0.3), 0.5)$	-2.2 [-2.7;-1.7]
$p^{\mu}_{\mathrm{PMQ+}}$	Population mean of reinfection mixture weights (PMQ+)		$\mathcal{N}(\mathrm{logit}(0.95), 0.25)$	3.5 [3.2;3.9]
$p^{\sigma}_{\mathrm{AS/CQ}}$	Population standard deviation of reinfection mixture weights (no PMQ)		$\mathcal{E}(1)$	2.5 [1.7;3.6]
$p^{\sigma}_{\mathrm{PMQ+}}$	Population standard deviation of reinfection mixture weights (no PMQ)		$\mathcal{E}(1)$	0.2 [0.1;0.7]
c^{AS}, c^{CQ}, $c^{\mathrm{PMQ+}}$	Mixture weights: recrudescence versus relapse (AS, CQ, and PMQ+, respectively)	yes	$\mathcal{N}(\mathrm{logit}(0.01), 0.25)$	-4.9 [-5.3;-4.4], -4.8 [-5.3;-4.4], -4.6 [-5.1;-4.1]

Supplementary Table 1: Summary of the prior distributions used to fit Model 2 to the pooled time-to-event data. †All mixture weight parameters (taking values between 0 and 1) were logit transformed and priors were specified on the logit scale. CI: credible interval. AS: artesunate monotherapy; CQ: chloroquine monotherapy; PMQ+: high-dose primaquine plus partner drug. $\mathcal{N}(\mu, \sigma)$ denotes the normal distribution with mean μ and standard deviation σ. $\mathcal{G}(\mu, k)$ denotes the gamma distribution with shape μ and rate k. $\mathcal{E}(\lambda)$ denotes the exponential distribution with rate λ. The posterior medians [CIs] are given to a minimum of two significant digits. *For the individual weights we show the median weight (range of the weights).

	Observed BPD recurrences estimated to be failures (%)	Failure rate in BPD trial (%)
Prior estimate (time-to-event model)	Not applicable	5.0 (3.1-7.9)
Prior estimate (genetic model)	66	Not applicable
Posterior estimate (time-to-event model)	13.5 (10.1-17.3)	2.3 (1.3-3.6)
Posterior estimate (genetic model)	29.9 (29.1-30.9)	4.8 (4.7-4.9)
Posterior estimate (combined model)	19.4 (16.6-23.7)	3.0 (2.4-4.0)

Supplementary Table 2: Rates of failure (relapse or recrudescence) in the BPD study inferred using the time-to-event model alone, the genetic model alone, and the combined model. The prior estimates average over the prior distributions specified for the time-to-event and genetic models. The posterior estimates are weighted means with 95% credible intervals provided in parentheses. All estimates are expressed as percentages. As an aside, the effect of incorporating both time-to-event and genetic data on probabilities of relapse and reinfection based on either data type alone for all 186 recurrences (BPD and VHX) experienced by individuals with only one or two recurrences is shown in Supplementary Fig. 9.

Marker Name	Motif	Chr (accession.version)	Position in chr	Range (bp)
PV.3.502	AACGGATG	3 (NC_009908.2)	451099-451266	128-265
PV.3.27	AAAC	3 (NC_009908.2)	493193-493320	85-240
PV.ms8	CAA	12 (NC_009917.1)	2322323-2322520	222-306
PV.1.501	GGTGAGA	1 (NC_009906.1)	473732-473851	76-195
PV.ms1	GAA	3 (NC_009908.2)	451788-452022	228-246
PV.ms5	CCT	6 (NC_009911.1)	538574-538747	163-187
PV.ms6	TCC	11 (NC_009916.1)	1760680-1760920	213-255
PV.ms7	GAA	12 (NC_009917.1)	1185288-1185438	133-160
PV.ms16	ACA	9 (NC_009914.1)	1593216-1593453	153-308

Supplementary Table 3: Details pertaining to microsatellite markers; chr (chromosome), bp (base pair).

Number of additional markers successfully typed	0	1	3	4	5	6
Number of 710 episodes typed	346	1	1	4	11	347
Number of 494 recurrences typed	230	0	1	2	8	253
Number of 493 paired recurrences typed	230	0	1	2	8	253
Number of 487 paired recurrences typed and analysed	229	0	1	2	8	247
Number of recurrences classified as relapse	116	0	1	2	8	238
Number of recurrences classified as reinfection	89	0	0	0	0	5
Number of recurrences classified as uncertain	24	0	0	0	0	4

Supplementary Table 4: Counts of genotyped episodes partitioned by the number of additional markers successfully typed. Episodes reported in the second line include enrolment and recurrent episodes. In total, 100 (85 VHX, 15 BPD) of 216 (46%) genotyped enrolment episodes were genotyped at six additional markers; and 264 (249 VHX, 15 BPD) of 494 (53%) genotyped recurrent samples were genotyped at six additional markers. Of all 364 genotyped at additional markers, 95% (347 total, 317 VHX, 30 BPD) were successfully genotyped at all additional six; see above. Of those 258 that were recurrent and successfully genotyped at one or more additional markers 97% (249 total, 240 VHX, 9 BPD) were classified as relapse; see above. This might suggest that additional markers positively bias inference of relapse. However, recurrences genotyped at additional makers were predominately obtained from patients in the VHX trial (249 VHX, 15 BPD). They are thus more likely relapses due to treatment obtained. To properly understand the effect of additional markers on relapse inference, we partitioned the relapse probability inferred in the null genetic data by the number of markers used to estimate the probability of relapse. Additional makers do not favourably bias relapse inference: the median probability of relapse decreases from the prior with one to three markers, stabilising around 0.25 thereafter (Supplementary Fig. 5).

	Time-to-event model assumption
1	No selection bias from early drop-out: i.e. censoring is non-informative. This may not hold as individuals who are at a higher risk of relapse (e.g. young males) may also be at a higher risk of loss to follow-up.
2	No lengthening of intervals between consecutive relapses in absence of reinfection. Studies with long follow-up have shown that as the hypnozoite reservoir is depleted, the average time to relapse lengthens[1]. This pattern was also shown with experimental inoculation of *P. cynomolgi* infections in Rhesus monkeys[2].
3	Time to reinfection is described by a single exponential distribution (no seasonality).
4	Time to relapse is described by a mixture of a Weibull and an exponential distribution.
5	Time to recrudescence is described by a single exponential distribution.
6	Chloroquine and piperaquine have the same post-treatment prophylactic period. In reality piperaquine has on average a slightly longer terminal elimination half-life and provides longer protection from recurrent infections.
7	There is no overlap between recurrence states: relapse, recrudescence and reinfection are considered mutually exclusive events. Individuals with large hypnozoite burdens who relapse frequently throughout the follow-up period could be reinfected at the same time as they relapse. Both the time-to-event and the genetic models would, on average, tend to label such an event as a relapse (depending of course on the timing and the genetic signature). Therefore, relapses can hide reinfection events.

Supplementary Table 5: List of assumptions in the time-to-event model. Assumptions are listed in an order of subjectively viewed importance (impact on results).

Supplementary Figures

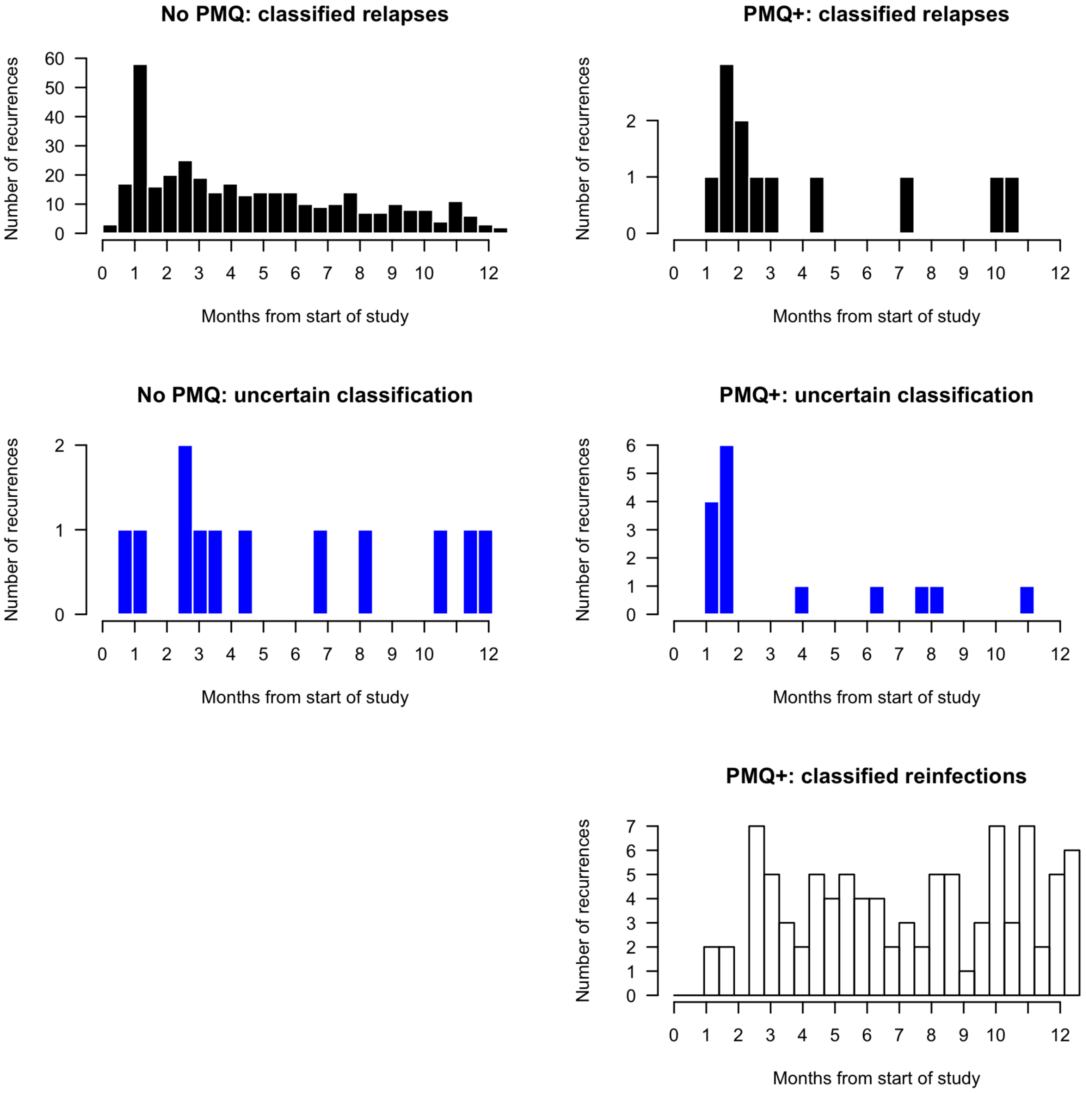

Supplementary Fig. 1: Histogram summary of the time-to-event distributions broken down by final recurrence state classification. None of the recurrences in the no primaquine (No PMQ) group were classified as reinfections. PMQ+ denotes recurrences following treatment with high-dose primaquine plus partner drug. We note that the distribution of times to relapse in No PMQ group is not representative of the overall distribution as recurrences were selectively genotyped and as such are not a representative sample.

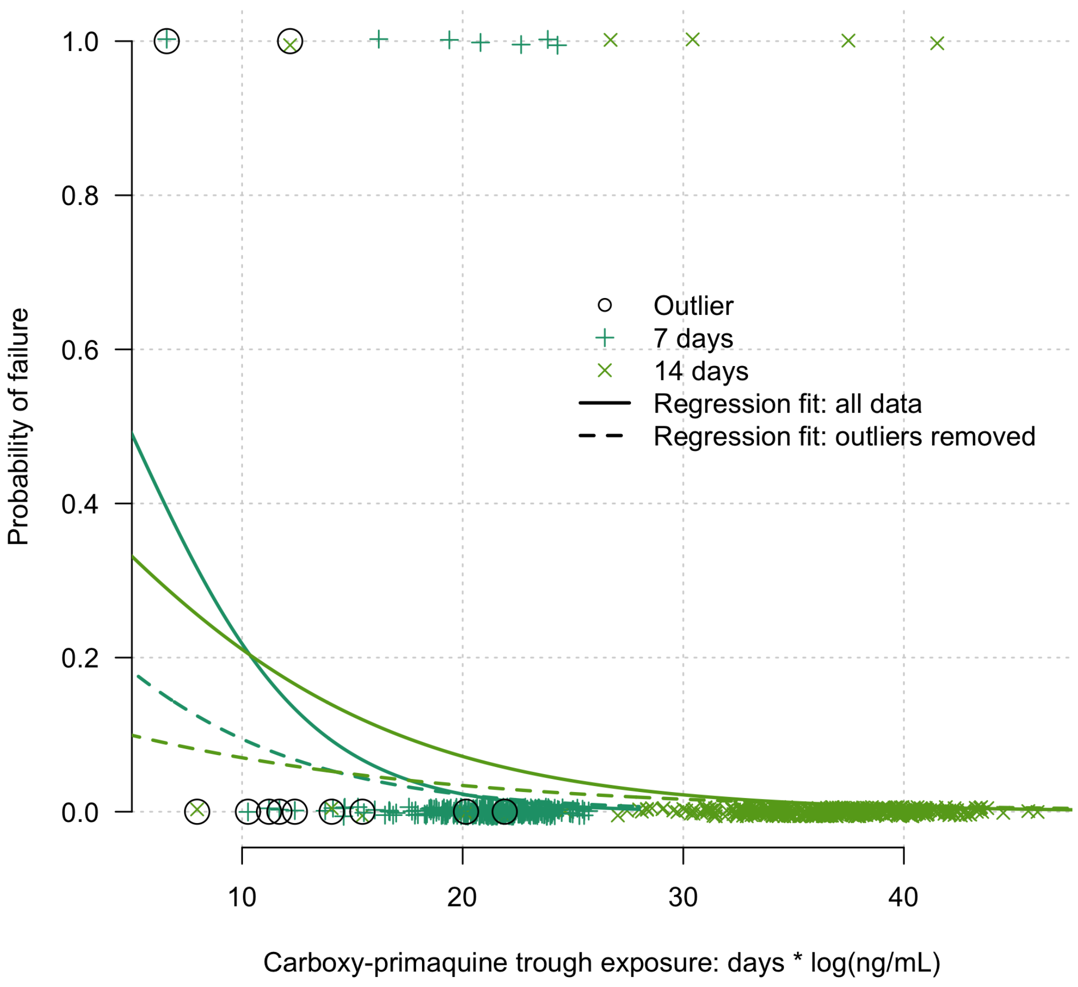

Supplementary Fig. 2: Estimated relationship between carboxy-primaquine exposure and primaquine failure. The proxy exposure to carboxy-primaquine is defined (lower bound) as the number of days of primaquine administration multiplied by the log trough concentration observed on day 7. The fitted trends using all the data are significant; they are shown by the thick lines. After removal of outliers (circles: defined as those episodes whose carboxy-primaquine trough concentrations were more than three standard deviations from the mean), the fitted trends (non-significant) are shown by the dashed lines.

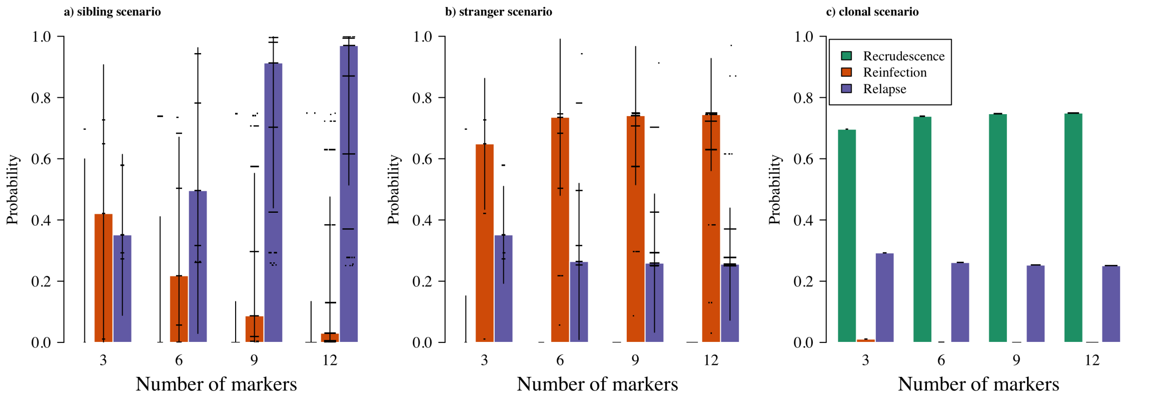

(i) Marker cardinality 4; non-erroneous data.

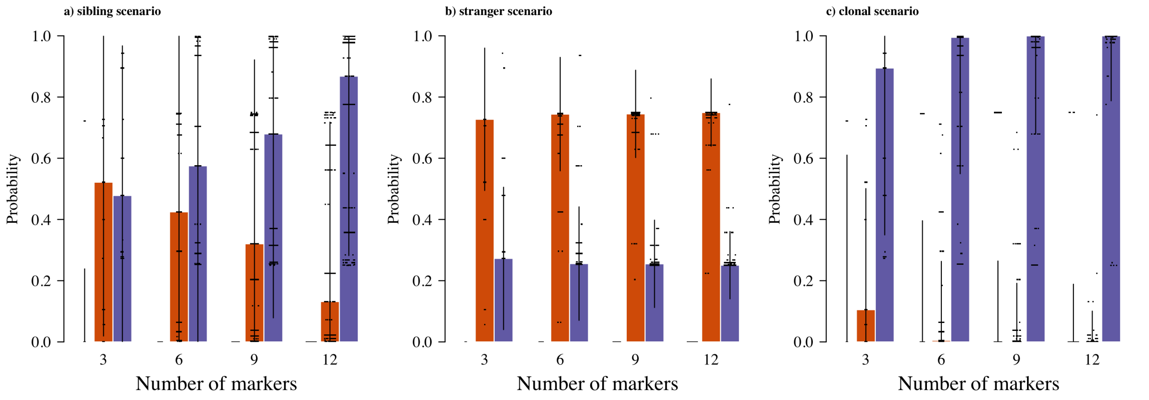

(ii) Marker cardinality 13; highly erroneous data.

Supplementary Fig. 3: Posterior probabilities of recurrence states as a function of the number of markers typed: the impact on recurrence state inference of lower effective cardinality (i) and highly erroneous data (ii). Coloured bars show the median of 250 posterior recurrent state probabilities (dots) with error bars extending ± two standard deviation. In all simulations, the COI was one in both the primary and recurrent episode. See main text Fig. 5 for an explanation of the expected behaviour under each scenario. In (i) markers were simulated with cardinality 4. In comparison with main text Fig. 5 (cardinality 13), more markers are required for probabilities to converge to expections. This is especially apparent under the sibling scenario. In comparison with main text Fig. 5, more markers are also required in the presence of genotyping error (ii). Genotyping error severely impacts inference under the clonal scenario: clones are misinterpreted as siblings and the probability of relapse tends towards one. To clearly illustrate model behaviour, the probability of a genotyping error was set extremely high (0.2 versus a realistic error < 0.01[3]).

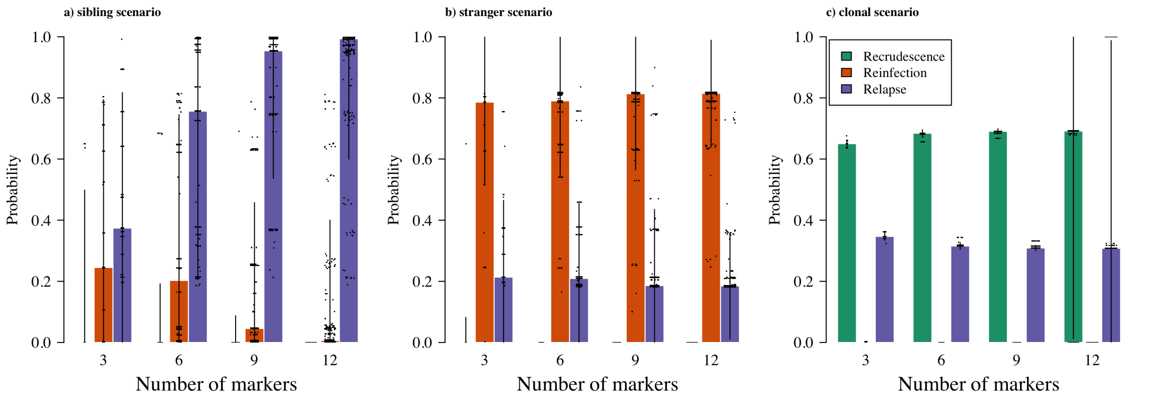

(i) COIs in the first and second infection of two and one respectively (COI 2-1)

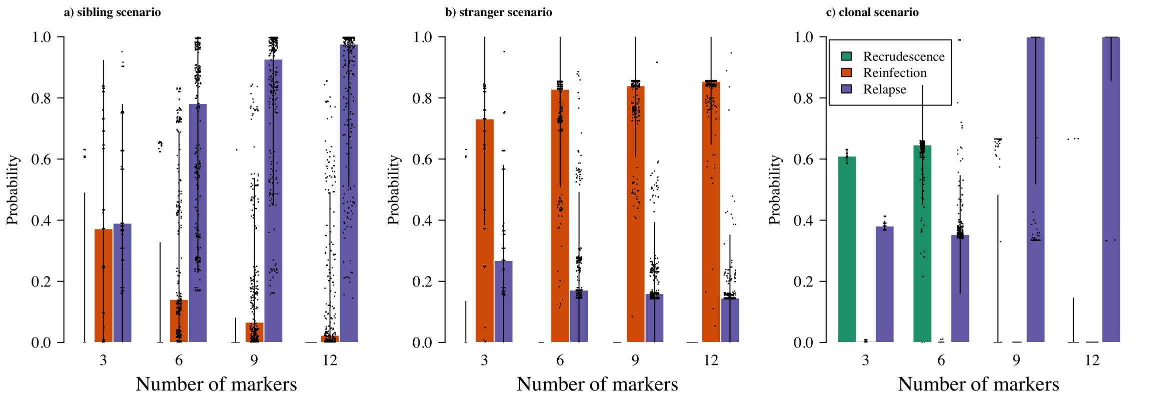

(ii) COIs in the first and second infection of three and one respectively (COI 3-1)

Supplementary Fig. 4: Posterior probabilities of recurrence states as a function of the number of markers typed: the impact on recurrence state inference, when the primary episode has COI of two (i) and three (ii). Coloured bars, dots and error bars as above (Supplementary Fig. 3). In all simulations, the cardinality of each marker was 13 and there was no genotyping error. See main text Fig. 5 for an explanation of expected behaviour under each scenario. Compared with main text Fig. 5, more markers are required for probabilities to converge. Additionally, (i) and (ii) illustrate the impact of failure to experimentally detect a minority parasite haploid genotype (a limitation of the data generating process), and failure to computationally detect a clonal haploid genotype in the observed data (a limitation of the genetic model). We illustrate these two failures using examples below. First, let us consider failure to experimentally detect a minority parasite haploid genotype: in the sibling COI 3-1 scenario (ii a) failure to detect the sibling parasite will result in the stranger COI 2-1 scenario (i b), thereby decreasing the probability of relapse, but not to zero. In the clone COI 3-1 scenario (ii c), failure to detect the clonal parasite will also result in the stranger COI 2-1 scenario (i b), thereby decreasing the probability of recrudescence to zero. Second, let us consider failure to computationally detect a clonal haploid genotype. This is a limitation of the genetic model due to the computational complexity. Its impact can be seen in the clonal COI 3-1 scenario (ii c). When data are highly diverse evidence of recrudescence (clonal phase) is liable to be overlooked because 1) the number of phasing combinations compatible with the observed data (A in the mathematical description of the genetic model; see Supplementary Methods) is huge and moreover grows exponentially with the number of markers, rendering evidence of recrudescence (clonal phase) a needle in an ever-growing haystack; and 2) since $\boldsymbol{H}_n^{(t)}$ in the genetic model is large, the genetic model implements probabilistic phasing which is liable to miss evidence of recrudescence (clonal phase) since A is huge (see Supplementary Methods). It is the fact that A is huge combined with probabilistic phasing that does not stabilise that leads to problems (alone, probabilistic phasing that does not stabilise does not present a problem). Moreover, this problem does not impact the analysis in the VHX and BPD data. In the VHX and BPD data, complex infections most likely derive from either relapsing or co-inoculated parasites (there is little opportunity for superinfection due to active follow up). As such, the parasites within real episodes are liable to be interrelated and thus less diverse.

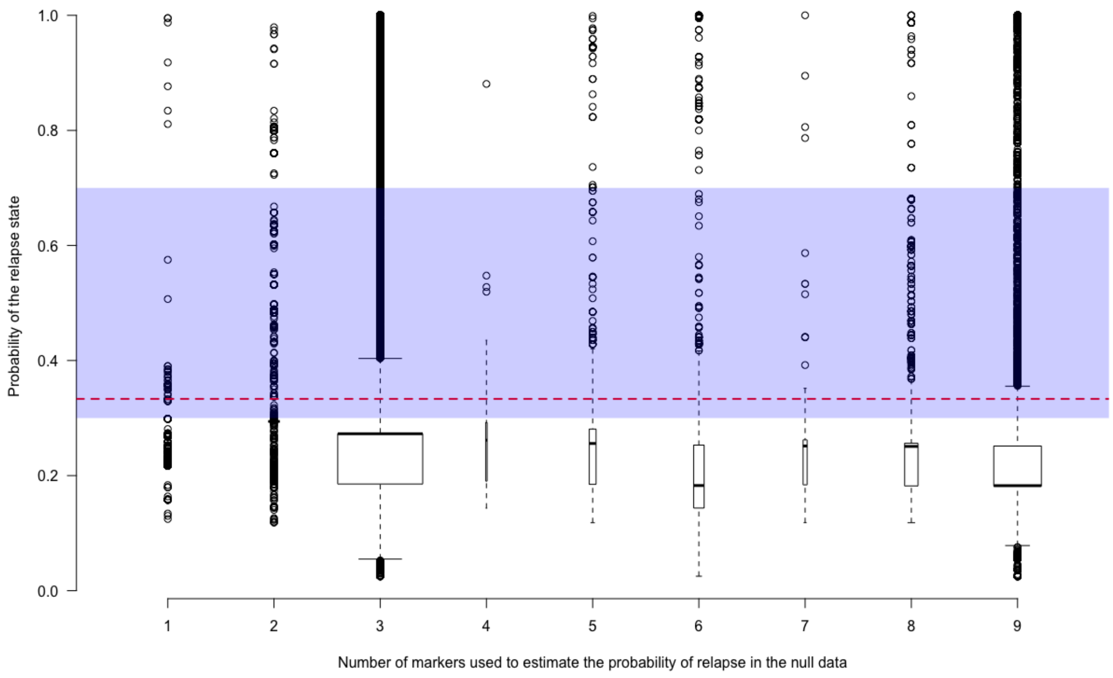

Supplementary Fig. 5: Boxplot showing the relationship between the number of shared genotyped markers used by the genetic model and the estimated probability of relapse in the null genetic data set (pairwise comparisons between episodes in different individuals). The prior probability of relapse used for these calculations is shown by the red dashed line (one third). The boxplot centre line marks the median; the vertical limits of each box mark the interquartile range; the whiskers extend to the most extreme data points within 1.5 times the interquartile range; all points outside that range are plotted. The width of the boxes is proportional to the square-root of the number of pairwise comparisons calculated (the majority of the comparisons are for 3 markers, $n = 177855$ comparisons, and for 9 markers, $n = 55759$).

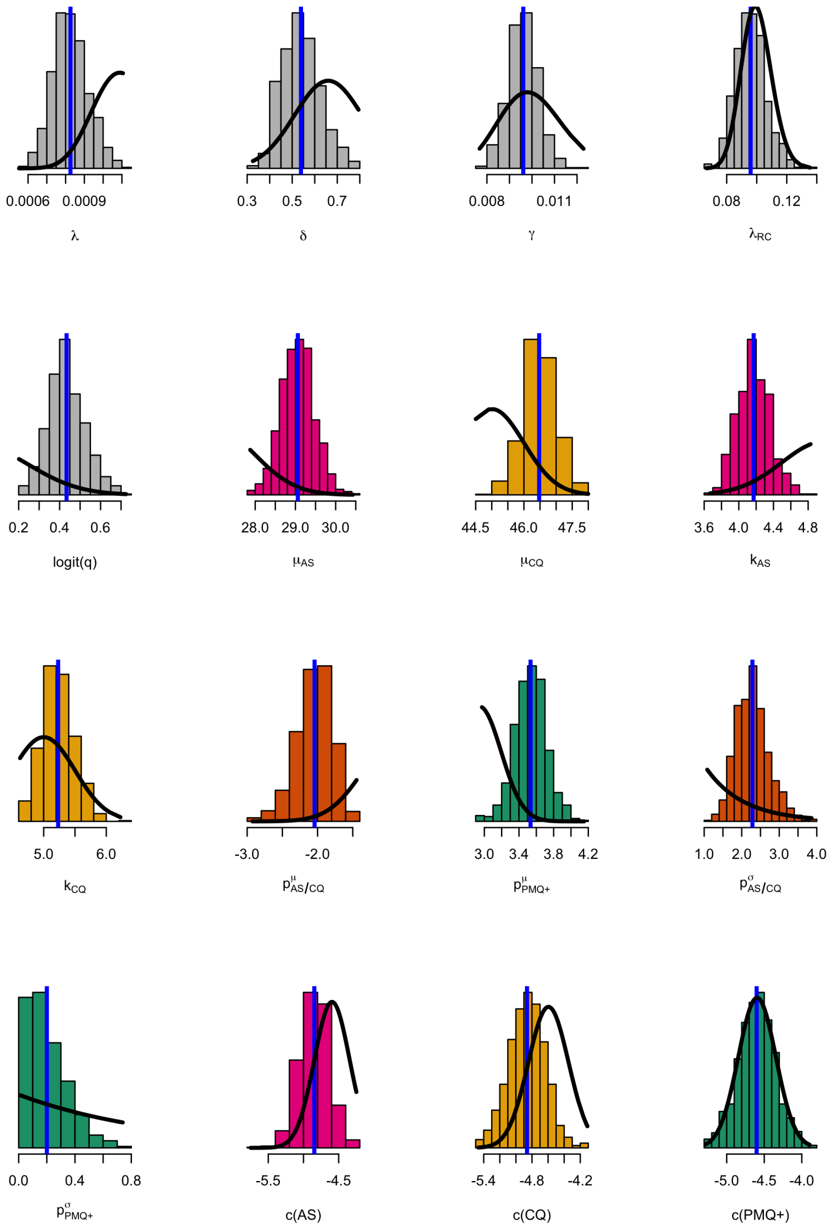

Supplementary Fig. 6: Prior and posterior comparison. The prior distributions are shown by the thick black lines. The posterior distributions are shown by the normalised histograms. Colors correspond to treatment specific parameters: grey is for global parameters; pink for artesunate monotherapy (AS) specific parameters; yellow for chloroquine monotherapy (CQ); green for primaquine plus partner drug (PMQ+); orange for non primaquine treated (AS and CQ combined). For each prior and posterior, the horizontal axis is in units of relative density. The vertical blue lines show the posterior mean values.

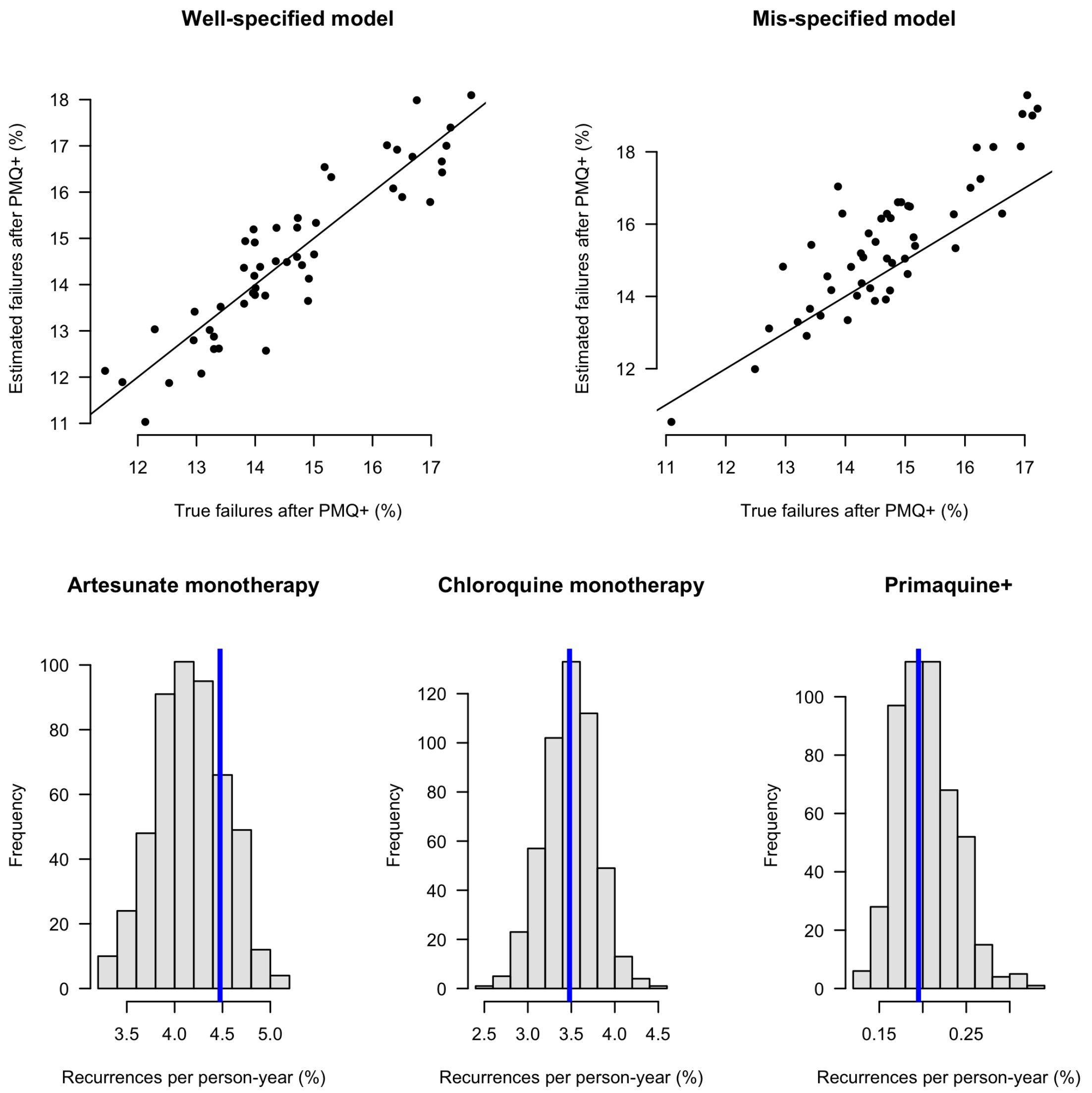

Supplementary Fig. 7: Simulation based model checking for the time-to-event model. The top two panels show the estimated percentage of failures (y-axis, relapse or recrudescence) against the true percentage of failures in 50 simulated data sets. The top-left panel shows this when data are simulated under the assumptions of the time-to-event model (well-specified model fit); the top-right panel shows this under model mis-specification whereby reinfection is seasonal. In this scenario, the model slightly overestimates the failure rate. The bottom three panels shows posterior predictive model checking whereby the summary statistics chosen to compute posterior predictive p-values are the recurrence rates per treatment arm. The histogram shows recurrence rates for 500 independent simulations under the assumptions of the time-to-event model where parameters are drawn from the posterior distribution of the model fit to the pooled data from the VHX and BPD studies. The blue vertical lines show the observed recurrence rates per person-year for each treatment arm in the VHX and BPD studies.

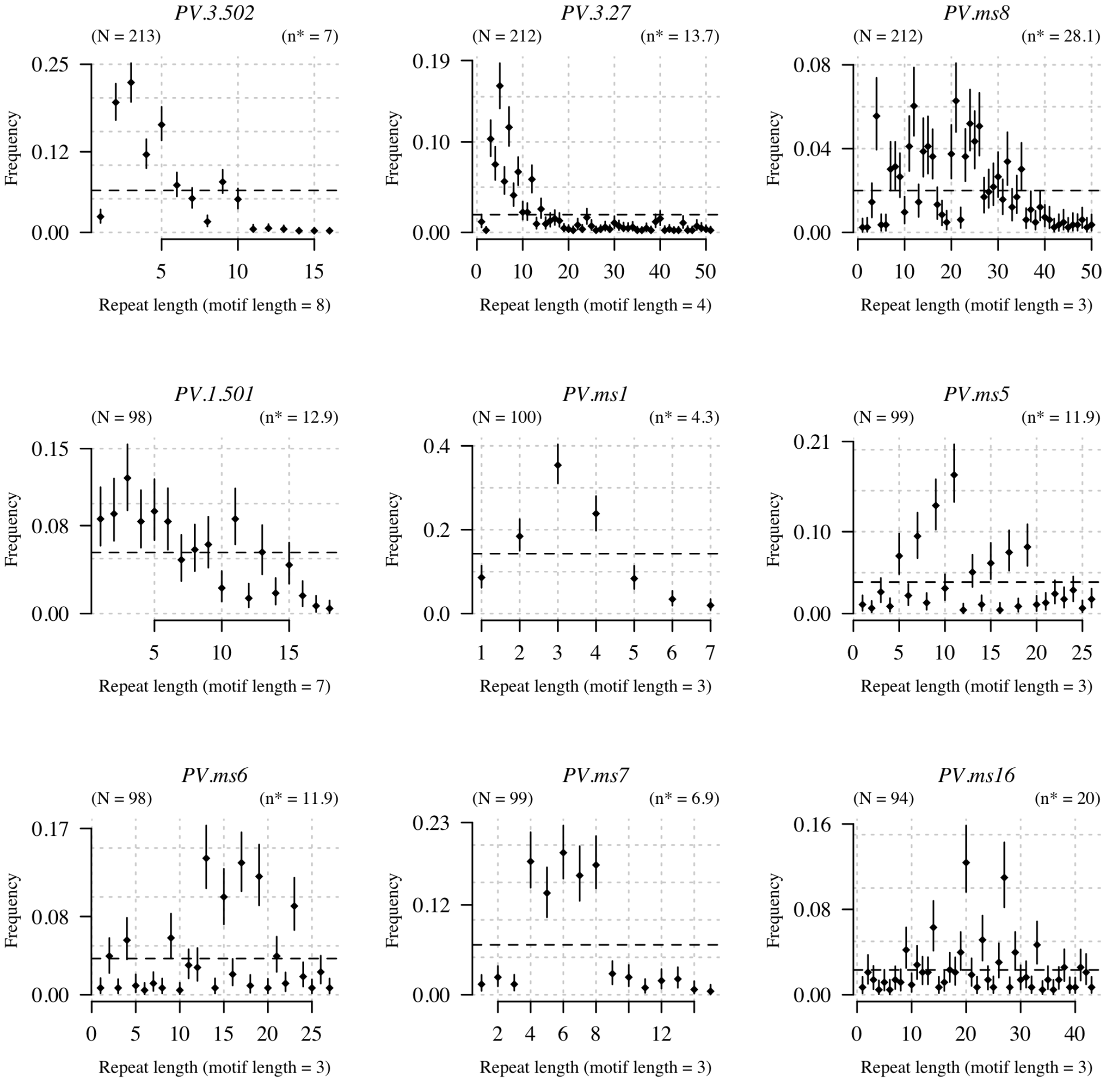

Supplementary Fig. 8: Allele frequencies and effective cardinality per microsatellite. For each microsatellite, frequencies were estimated using all available genetic data from enrolment episodes (79 BPD, 137 VHX) and a multinomial-Dirichlet model. The number of enrolment episodes typed for each microsatellite is denoted by N. The top row shows the microsatellites which were typed most frequently (PV.3.502, PV.3.27, PV.ms8). The estimated effective cardinality of each marker is denoted by n^*. Black circles depict posterior mean frequency estimates. Vertical lines depict 95% credible intervals (constructed using 1000 draws from the posterior distribution over allele frequencies). The elements of the prior vector (length equal to the maximum repeat length observed) were set to one, thereby interpolating unobserved alleles with intermediate repeat lengths. The dotted horizontal line shows the discrete uniform distribution over all alleles (including those with unobserved intermediate lengths).

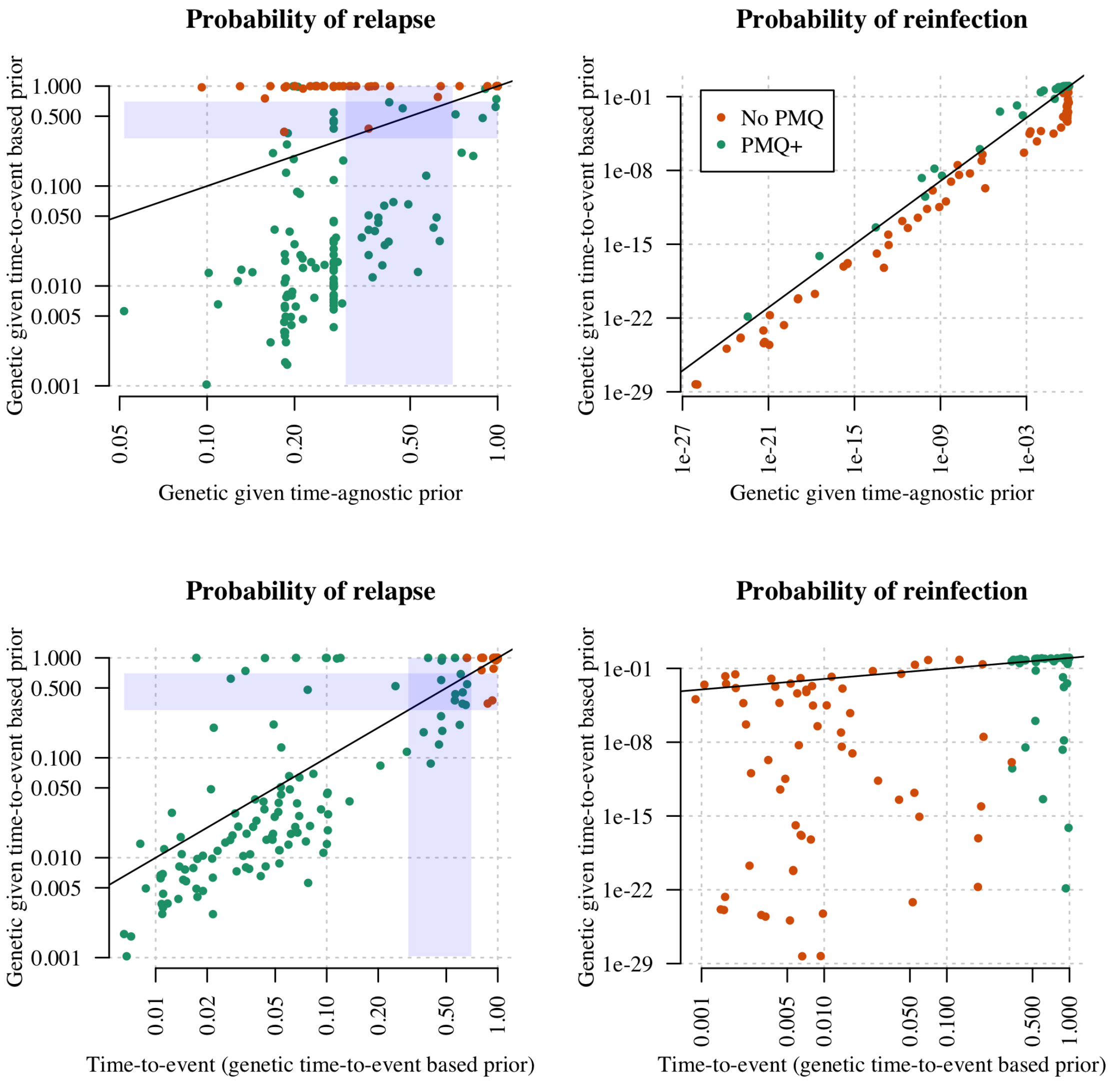

Supplementary Fig. 9: Effect of incorporating both time-to-event and genetic data on probabilities of relapse and reinfection based on either data type alone. The plots show results for all 186 recurrences (66 no PMQ, 120 PMQ+) experienced by individuals with only one or two recurrences. The top two plots show the effect of incorporating time-to-event data relative to genetic data alone on probabilities of relapse (left) and reinfection (right). The bottom two plots show the effect of incorporating genetic data relative to time-to-event data alone on probabilities of relapse (left) and reinfection (right). The diagonal line marks the line of equality. Blue bands mark the arbitrarily defined relapse classification 'uncertainty zones'. Points in top left and bottom right 'certainty zones' (non-blue rectangles) mark recurrences whose classification changes from not relapse to relapse and vice versa, respectively, upon incorporation of data. Upon incorporation of time-to-event data many 'No PMQ' were classified as relapse where they were not previously. Upon incorporation of genetic data, 13 'PMQ+' treated episodes are classified as relapses where zero were classified as relapse previously (top left plot).

Supplementary Methods

Descriptive overview and assumptions of the genetic model: To infer the probability of recurrence states, we construct a statistical model that exploits evidence of relatedness expected within a single mosquito inoculum following recombination. The only relationships between sporozoites that are exclusive to a single mosquito are those between meiotic clones and siblings (Supplementary Table 6). In other words, relapse can be caused by many types of sporozoites, including those which are strangers in relation to one another, but, in the absence of recrudescence, only meiotic clones and meiotic siblings provide conclusive evidence of relapse (Supplementary Table 6). Genetic data cannot discern clones and siblings that are meiotic or not. We thus use a coarse definition of clones and siblings in this model, ignoring whether the relationship is meiotic. We use the term stranger to refer to all parasites whose shared ancestry dates back beyond the most recent mosquito inoculation. Consequently, the term clone refers to first-generation clones only. We assume zero probability of reinfection with first-generation clones or siblings, since the probability of a mosquito feeding on the same human host consecutively is low. Importantly, we do allow strangers that are genetically identical by chance. This captures some clones resulting from clonal expansion (i.e. multiple generations of selfing). Parent-offspring pairs are considered siblings under our model since they have an expected relatedness equal to 0.5 in the absence of inbreeding. We ignore half-siblings whose expected relatedness is 0.25 in the absence of inbreeding. However, we do allow half-siblings in the sense that we allow two or more alleles per locus within infections assumed to contain only siblings (i.e. we allow for collections of siblings that together share more than two parents). The main model assumptions (including those mentioned above, also summarised in Supplementary Table 6) are as follows.

1. In relation to one another, sporozoites within an inoculum are either clones, siblings or strangers with respective expected relatedness of 1, $0.5 + \alpha$ and $0 + \alpha$ (where $\alpha = 0$ in the absence of inbreeding, otherwise $\alpha \in (0, 0.5]$)
2. Recrudescence has non-zero probability if and only if all parasites are clones of those in the directly preceding infection
3. No genotyping errors (i.e. no microsatellite slippage)
4. No mutation (i.e. all diversity due to standing variation)
5. 100% detection of all parasite clones during patent infection
6. The complexity of a given infection (COI) is equal to the maximum number of distinct alleles seen at any microsatellite within that infection
7. The probability of being reinfected with parasites that share alleles with previously inoculated parasites is equal to that of identity by chance (i.e. the product of the frequencies of the alleles observed).
8. Mutually exclusive recurrence states
9. All microsatellites are independent and neutral

Assumption 1 disregards half-siblings. However, as described above, within infections assumed to contain only siblings, we allow more than two alleles per locus. Half-siblings are possible within

an inoculum if both i) a mosquito takes a blood meal from an infection with COI ≥ 3 and ii) two or more zygotes derived from the cross fertilisation of three or more genetically distinct gametes survive the severe bottleneck of successful oocyst formation[4,5]. Given the severe bottleneck, and the fact that only 30 of the 710 (4%) genotyped episodes in the pooled dataset have COI estimates ≥ 3, half-siblings are likely to be rare in the data analysed here. If any are present, the likelihood of the observed alleles will be misspecified with expected IBD=0.5 under the model.

Assumption 1 also states that all siblings have the same expected relatedness (0.5 in the absence of inbreeding). In general, this assumption holds for all full siblings, meiotic or not. However, if we condition on meiotic siblings having one or more genetic difference, the expected relatedness between two meiotic siblings is 0.33 (see Supplementary Note). Within infections with COI≥ 2, we collapse clonal relationships between haploid parasite genotypes. This means that the expected relatedness is over-specified (by 0.2) if an infection contains meiotic sibling parasites with distinct haploid genotypes. This level of misspecification is likely inconsequential given natural variation introduced by meiosis[6,7]. Moreover, if we average over the probability that the two distinct parasites came from different oocysts, the extent of misspecification rapidly declines.

To allow for inbreeding under the model, we have included the parameter $\alpha \in [0, 0.5]$ as specified in Assumption 1. A recent study found compelling evidence of inbreeding and selfing in oocysts from mosquitoes fed on *P. vivax* blood samples from the Thailand-Myanmar border in 2013[8]. As a sensitivity analysis, we reran the computations with $\alpha = 0.175$ (see github notebook) and compared with results for $\alpha = 0$. The overall results are robust to this change, the only impact being that some primaquine treated recurrences have a lower probability of relapse.

Assumptions 2 to 6 render inference of recrudescence frail under the model. Given there is very little evidence of *P. vivax* antimalarial resistance on the Thailand-Myanmar border, this likely has little impact on our results. The model would require modification before application to data from a region where *P. vivax* antimalarial resistance is suspected, however. In contrast, inference of relapse is relatively robust to these assumptions. This is because we account for relapses caused by siblings under the model; see Supplementary Fig. 3 and 4. Conditioning on sibling relatednesss (IBD=0.5) absorbs differences caused by mutation and slippage, especially since within infections assumed to contain only siblings we allow more than two alleles per locus.

Assumption 7 is likely to hold except in the rare event that an infected mosquito consecutively feeds on the same human host (thereby transmitting a first-generation clone or recombinant offspring to the same human host from whom the parental parasites were sourced).

Assumption 8 implies that all polyclonal reinfections are generated by co-inoculation. That is, we do not allow coincidental blood-stage parasites from different mosquito inoculations unless they are hynozoite-derived. Both the BPD and VHX trials had active follow-up and all asymptomatic infections were treated so this assumption is likely to hold. It would not hold in the context of passive detection or untreated asymptomatic infections. This assumption also implies that we might miss reinfections in some individuals with frequent sibling or clonal relapses.

Neutrality of microsatellites (assumption 9) implies that allele frequencies of hypnozoites are the same as those in the wider population. The microsatellite markers used in this study were designed specifically to meet this assumption[9,10]. Future data types may include non-independent markers. Extension of the model to capture linkage between non-independent markers is possible (see[7,11,12]).

		Recrudescence	Relapse	Reinfection
Real life	Meiotic clones	Yes	Yes	No
	Meiotic siblings	No	Yes	No
	Clones	No	Yes	Yes but unlikely
	Siblings	No	Yes	Yes but unlikely
	Half-siblings	No	Yes	Yes but unlikely
	Parent-offspring	No	Yes	Yes but unlikely
	Strangers*	No	Yes	Yes
Model	Clones†	Yes	Yes	No
	Siblings‡	No	Yes	No
	Strangers*	No	Yes	Yes

Supplementary Table 6: Compatibility of parasite relationships *across* blood-stage infections conditional on recrudescence, relapse and reinfection assuming all parasites are detected during patent infection (i.e. underlying truth). Meiotic clones are parasites derived from a single selfed oocyst, which means they must have been coinoculated. Meiotic siblings are parasites derived from a single outcrossed oocyst, which also means they must have been coinoculated. Clones, siblings, parent-offspring and half-siblings are all parasites that share common gamete genotypes but are from different oocysts. They can occur in a single mosquito or in different mosquitoes (e.g. contemporaneous mosquitoes that share a common human source, or sequential mosquitoes linked to a common human host who acts a source to the first mosquito and sink to the second). Strangers* include all parasites whose shared common ancestry dates back beyond the most recent mosquito. Under the model, clones† includes both meiotic and not; siblings‡ includes parent-offspring and meiotic and not, but excludes half-siblings in the sense that they have expected relatedness of 0.25 (in the absence of inbreeding).

Mathematical description of the genetic model: In the following text we provide a full mathematical description of the genetic model in four parts: *Model overview, Evaluation of the prior, Evaluation of the likelihood and Model implementation.* A running example is provided in boxes. The full set of mathematical notation used in this model is given in Supplementary Table 7.

***

Model overview: This Bayesian model estimates the probability distribution over the possible recurrence states $R_n^{(t)}$, for recurrence t experienced by individual n, given all available genetic data for that individual, denoted $\boldsymbol{y}_n$. The Bayesian posterior probability of the relapse state L for the tth recurrence experienced by the nth individual can be written as:

$$\mathbb{P}\left(R_n^{(t)} = L \mid \boldsymbol{y}_n\right) = \sum_{\mathcal{L}} \left\{ \frac{\mathbb{P}(\boldsymbol{y}_n \mid \boldsymbol{R}_n)\,\mathbb{P}(\boldsymbol{R}_n)}{\sum_{\text{All } \boldsymbol{R}_n} \mathbb{P}(\boldsymbol{y}_n \mid \boldsymbol{R}_n)\,\mathbb{P}(\boldsymbol{R}_n)} \right\}, \tag{1}$$

where

- $\mathbb{P}(\boldsymbol{y}_n \mid \boldsymbol{R}_n)$ denotes the likelihood and $\mathbb{P}(\boldsymbol{R}_n)$ denotes the prior;
- $\boldsymbol{R}_n = \left(R_n^{(1)}, \ldots, R_n^{(T_n)}\right)$ where $R_n^{(t)} \in \{C, L, I\}$ for $t > 0$ where C, L, I denote recrudescence, relapse and reinfection, respectively, and T_n is the total number of recurrences experienced by the nth individual (s.t. $\boldsymbol{R}_n \in \{C, L, I\}$ if $T_n = 1$ and $\boldsymbol{R}_n \in \{II, LL, CC, IC, CI, IL, LI, LC, CL\}$ if $T_n = 2$);
- $\mathcal{L} = \left\{\boldsymbol{R}_n : R_n^{(t)} = L\right\}$ (i.e. $\mathcal{L}$ is the set of all $\boldsymbol{R}_n$ such that $R_n^{(t)} = L$);
- $\boldsymbol{y}_n = \left(\boldsymbol{y}_n^{(1)}, \ldots, \boldsymbol{y}_n^{(T_n)}\right)$ where $\boldsymbol{y}_n^{(t)} = \left(\boldsymbol{y}_{nm}^{(t)}, \ldots, \boldsymbol{y}_{nM}^{(t)}\right)$ for $t = 0, \ldots, T_n$, and for $m = 1, \ldots, M$ microsatellites typed, $\boldsymbol{y}_{nm}^{(t)} = \left\{y_{nm}^{(t)}\right\}$ is the set of alleles observed at the mth microsatellite typed in the tth infection experienced by the nth individual (e.g. $\boldsymbol{y}_{n3}^{(2)} = \{1, 3\}$ in Equation (2)).

Example per-person set of data, $\boldsymbol{y}_n$

$$\boldsymbol{y}_n = \begin{pmatrix} 2,8 & 2 & 8 \\ 7,4 & 3 & 9 \\ 3 & 6 & 1,3 \end{pmatrix} \begin{matrix} \text{sets of alleles detected at the first microsatellite, } m = 1, \\ \text{sets of alleles detected at the second microsatellite, } m = 2, \\ \text{sets of alleles detected at the third microsatellite, } m = 3. \end{matrix} \tag{2}$$

(columns: $t = 0$, $t = 1$, $t = 2$)

***

Evaluation of the prior: The prior, $\mathbb{P}(\boldsymbol{R}_n)$ where $\boldsymbol{R}_n = \left(R_n^{(1)} \ldots, R_n^{(T_n)}\right)$, is evaluated by modelling each $R_n^{(t)}$ as a random variable from a categorical distribution over C, L and I with

$n = 1, \dots, N$	index over N individuals
$m = 1, \dots, M$	index over M microsatellites
$t = 0, \dots, T_n$	index over infections for the nth individual, where t = 0 indexes the enrollment episode
T_n	total number of recurrent infections experienced by the nth individual
$y^{(t)}_{n_m}$	a single allele detected at the mth microsatellite of the nth individual's tth infection
$\boldsymbol{y}^{(t)}_{n_m} = \left\{ y^{(t)}_{n_m} \right\}$	set of alleles detected at the mth microsatellite of the nth individual's tth infection
$\boldsymbol{y}^{(t)}_n = \left(\boldsymbol{y}^{(t)}_{n_1}, \dots, \boldsymbol{y}^{(t)}_{n_M} \right)$	set of alleles detected in the nth individual's tth infection
$\boldsymbol{y}_n = \left(\boldsymbol{y}^{(0)}, \dots, \boldsymbol{y}^{(T_n)} \right)$	all genetic data available for the individual n (e.g. Equation (2))
$c^{(t)}_n = \max \left(\mid \boldsymbol{y}^{(t)}_{n1} \mid, \dots, \mid \boldsymbol{y}^{(t)}_{nM} \mid \right)$	complexity of infection of the tth infection of the nth individual (assumed not random)
$R^{(t)}_n \in \{C, L, I\}$	recurrence state (C if a recrudescence, L if a relapse, and I if a reinfection) of the tth recurrence of the nth individual
$\boldsymbol{R}_n = \left(R^{(1)}_n, \dots, R^{(T_n)}_n \right)$	recurrence states for $t = 1, \dots, T_n$ recurrences experience by the nth individual
$\boldsymbol{H}^{(t)}_n$	matrix of haploid genotypes compatible with $\boldsymbol{y}^{(t)}_n$
$\hat{\boldsymbol{\pi}}^{(t)}_n = \left(\hat{\pi}^{(t)}_{n_C}, \hat{\pi}^{(t)}_{n_L}, \hat{\pi}^{(t)}_{n_I} \right)$	individual prior probability point estimates of recurrence states (derived from the time-to-event model)
IBD $\in \{0, 1\}$	hidden IBD state (1 denotes IBD, 0 denotes not IBD)
$\mathbb{I}_{\Omega}(x)$	indicator function equal to one if $x \in \Omega$ and 0 otherwise
$\|\Omega\|$	size (i.e. number of elements) of a set Ω
$\boldsymbol{G}_{n_{ab}} = \left(\boldsymbol{E}_{n_{ab}}, \boldsymbol{V}^{(0)}_{n_{ab}}, \dots, \boldsymbol{V}^{(T_n)}_{n_{ab}} \right)$	graph of relationships between parasite haploid genotypes across all infections of the nth individual
$\|\boldsymbol{G}_{n_{ab}}\| = \sum_{t=0}^{T_n} c^{(t)}_n$	size (number of vertices) of $\boldsymbol{G}_{n_{ab}}$ (depends on $\boldsymbol{y}_n$ via $c^{(t)}_n$)
$i, j = 1, \dots, \mid \boldsymbol{G}_{n_{ab}} \mid$	indices over vertices and edges in graphs
$\mathcal{I}^{(t)}$	set of indices over vertices and edges within the tth infection: $\left\{ 1, \dots, c^{(t)}_n \right\} + \mathbb{I}_{t>0}(t) \sum_{z=0}^{t-1} c^{(z)}_n$
$\boldsymbol{E}_{n_{ab}} = \left\{ e_{n_{ab_{ij}}} \right\}$	set of all edges in $\boldsymbol{G}_n$ where $j < i$ and $i = 2 \dots \mid \boldsymbol{G}_{n_{ab}} \mid$
$\boldsymbol{V}^{(t)}_{n_{ab}} = \left\{ v_{n_{ab_i}} \right\}$	set of vertices for the tth infection in $\boldsymbol{G}_{n_{ab}}$ where $i \in \mathcal{I}^{(t)}$
$a = 1, \dots, A$	index over labelled graphs that differ with respect to their vertex haploid genotype labels, where A is the number of ways to label a graph (disregarding edge relationship labels) with vertex haploid genotype labels compatible with $\boldsymbol{y}_n$ (i.e. only includes graphs whose probability is non-zero according to Equation (7))
$b = 1, \dots, B$	index over labelled graphs that differ with respect to their edge relationship labels, where B is the number of viable ways to label a graph (disregarding vertex haplotype genotype labels) with edge relationship labels. Viable graphs are those that obey the transitive property and, additionally, have no clonal edges within an infection, see section entitled *Viable graph brute-force search algorithm*)
$\boldsymbol{h}_{n_{ab_i}} = \left(h_{n_{ab_{i_1}}}, \dots, h_{n_{ab_{i_M}}} \right)$	vertex haploid genotype label of vertex $v_{n_{ab_i}}$, where $h_{n_{ab_{i_m}}} \in \boldsymbol{y}^{(t)}_{n_m}$ for all $i \in \mathcal{I}^{(t)}$
$k_{n_{ab_{ij}}} \in \{\text{str, sib, clo}\}$	relationship (a.k.a. kinship) label of edge $e_{n_{ab_{ij}}}$
sib	sibling relationship between a pair of parasites
str	stranger relationship between a pair of parasites
cln	clonal relationship between a pair of parasites
f_{i_m}	frequency of the allele at the mth microsatallite of the haploid genotype on the ith vertex of $\boldsymbol{G}_{n_{ab}}$
$\alpha \in [0, 0.5]$	additive effect on $\mathbb{P}(\text{IBD} \mid k_{n_{ab_{ij}}})$ of background population-level inbreeding

Supplementary Table 7: Mathematical notation in genetic model. In the top division we list non-graph notation and the subscript n is dropped where fixed. In the bottom division we list graph notation and subscripts n, a and b are dropped where fixed.

per person recurrence probability point estimates generated under the time-to-event model, namely $\hat{\boldsymbol{\pi}}_n^{(t)} = \left(\hat{\pi}_{n_C}^{(t)}, \hat{\pi}_{n_L}^{(t)}, \hat{\pi}_{n_I}^{(t)}\right)$, for $t = 1, \ldots, T_n$,

$$\mathbb{P}(\boldsymbol{R}_n) = \prod_{t=1}^{T_n} \hat{\pi}^{(t)}_{n_{R_n^{(t)}}}. \tag{3}$$

***

Evaluation of the likelihood: The likelihood, $\mathbb{P}\left(\boldsymbol{y}_n \mid \boldsymbol{R}_n\right)$, is evaluated by summing over person-specific (indexed by n) vertex labelled (indexed by a) and edge labelled (indexed by b) graphs of relationships over parasite haploid genotypes within and across infections, $\boldsymbol{G}_{n_{ab}}$,

$$\mathbb{P}\left(\boldsymbol{y}_n \mid \boldsymbol{R}_n\right) = \sum_{a=1}^{A}\sum_{b=1}^{B} \mathbb{P}\left(\boldsymbol{y}_n \mid \boldsymbol{G}_{n_{ab}}\right)\mathbb{P}\left(\boldsymbol{G}_{n_{ab}} \mid \boldsymbol{R}_n\right). \tag{4}$$

The solution to Equation (4) is described as follows in three steps: first we describe the set of fully labelled graphs $\boldsymbol{G}_{n_{ab}}$, second we describe $\mathbb{P}\left(\boldsymbol{y}_n \mid \boldsymbol{G}_{n_{ab}}\right)$, and third we describe $\mathbb{P}\left(\boldsymbol{G}_{n_{ab}} \mid \boldsymbol{R}_n\right)$. The individual, n, is considered fixed throughout the next sections so the subscript n is dropped from $\boldsymbol{y}_n$, $\boldsymbol{G}_{n_{ab}}$, $\boldsymbol{R}_n$ and T_n, etc.

*

Step one considers vertex and edge labelled graphs of parasite relationship. Let the following $\boldsymbol{G}_{ab} = \left\{\boldsymbol{E}_{ab}, \boldsymbol{V}_{ab}^{(0)}, \ldots, \boldsymbol{V}_{ab}^{(T)}\right\}$ denote an undirected, edge and vertex labelled, viable graph of relationships over parasite haploid genotypes within and across $t = 0, \ldots, T$ infections for a given individual. a is an index over all the possible combinations for labelling the vertices; b is an index over all the possible combinations for labelling the edges; viable graphs include only those that obey the transitive property and have no clonal edges within an infection (see paragraph on **Viable graph brute-force search algorithm**). $\forall a \in \{1 \ldots A\}, b \in \{1 \ldots B\}$, we define the following:

- $\boldsymbol{E}_{ab} = \left\{e_{ab_{ij}}\right\}_{j=1,i=2}^{j<i,\ldots,|G_{ab}|}$, the set of all edges in $\boldsymbol{G}_{ab}$;
- $\boldsymbol{V}_{ab}^{(t)} = \{v_{ab_i}\}_{i\in\mathcal{I}^{(t)}}$, the set of all vertices in $\boldsymbol{G}_{ab}$ corresponding to the tth infection, where $\mathcal{I}^{(t)} = \{1, \ldots, c^{(t)}\} + \mathbb{I}_{t>0}(t)\sum_{z=0}^{t-1} c^{(z)}$ is the set of indices corresponding to the tth infection and $c^{(t)}$ is the COI of the tth infection;
- The relationship (a.k.a. "kinship") label $k_{ab_{ij}} \in \{\text{str}, \text{sib}, \text{cln}\}$, for each edge $e_{ab_{ij}}$, where str, sib and cln denote stranger, sibling and clone, respectively;
- The haploid genotype label $\boldsymbol{h}_{ab_i} = \left(h_{ab_{i_1}}, \ldots, h_{ab_{i_M}}\right)$ for each vertex, v_{ab_i}, where $h_{ab_{i_m}} \in \boldsymbol{y}_{n_m}^{(t)}$ for $i \in \mathcal{I}^{(t)}$, $t = 0, \ldots, T$, and $m = 1, \ldots M$.

Since we assume that the COI of the tth infection, $c^{(t)}$, is equal to the maximum number of distinct alleles seen at any microsatellite within that infection, i.e. $c^{(t)} = \max(|\boldsymbol{y}_1^{(t)}|, \ldots, |\boldsymbol{y}_M^{(t)}|)$, and since we assume no genotyping error nor mutation (assumptions 3 and 4), for $\mathbb{P}\left(\boldsymbol{y} \mid \boldsymbol{G}_{ab}\right) > 0$

Example graphs compatible with $\boldsymbol{y}$ of Equation (2).

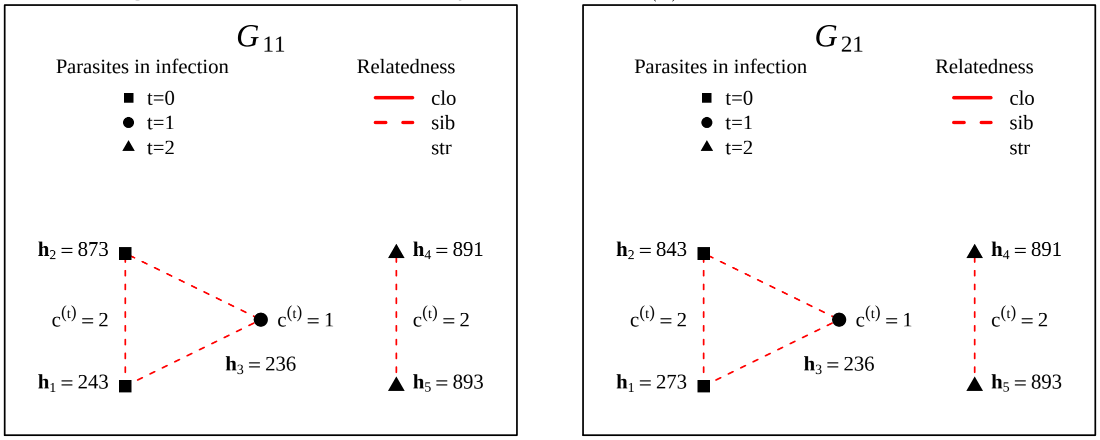

Supplementary Fig. 10: Within each example graph, $\boldsymbol{G}_{ab}$ where $a \in \{1, 2\}$ and $b = 1$, indices a and b are fixed so dropped hereafter and within each subplot above (besides the graph name, $\boldsymbol{G}_{ab}$). The vertices of both graphs are labelled from left to right: $\boldsymbol{V}^{(0)} = \{v_1, v_2\}$ with haploid genotype labels $\{\boldsymbol{h}_1, \boldsymbol{h}_2\}$, $\boldsymbol{V}^{(1)} = \{v_3\}$ with haploid genotype label $\boldsymbol{h}_3$, and $\boldsymbol{V}^{(2)} = \{v_4, v_5\}$ with haploid genotype labels $\{\boldsymbol{h}_4, \boldsymbol{h}_5\}$. 'Parasites' is shorthand for parasite haploid genotypes. The edge relatedness (a.k.a. kinship) labels of both graphs are the same: $k_{12} = sib$, $k_{23} = sib$, $k_{13} = sib$ and $k_{45} = sib$, while the rest are all str, such that $b = 1$ for both graphs. On the contrary, the vertex haploid genotype labels $\boldsymbol{h}_1$ and $\boldsymbol{h}_2$ differ across the example graphs and so the example graphs have different indices, $a = 1$ and $a = 2$.

we require that $\left|\boldsymbol{V}_{ab}^{(t)}\right| = c^{(t)}$ for all t (i.e. the number of vertices must equal the COI) and all alleles in $\boldsymbol{y}$ need to be represented at least once by the vertex haploid genotype labels. Supplementary Fig. 10 shows two example graphs for the example set of data in Equation (2). The graphs in Supplementary Fig. 10 differ in their vertex haploid genotype labels $\boldsymbol{h}_{ab_1}$ and $\boldsymbol{h}_{ab_2}$, but both have the same set of edge relationship labels, $\left\{k_{ab_{ij}}\right\}$ for all $j < i$ and $i = 2, \ldots, |\ \boldsymbol{G}_{ab}\ |$. As mentioned above, we use a and b to index over vertex haploid genotype labels and edge relatedness labels, respectively, and so the two graphs $\boldsymbol{G}_{ab}$ of Supplementary Fig. 10 have different $a \in \{1, 2\}$ but the same $b = 1$.

The number of ways to allocate haploid genotype labels to a graph for the nth individual, A, is enumerated independently of edge relatedness labels. A depends on both $|\boldsymbol{y}_m^{(t)}|$ (i.e. the number of alleles detected at a given microsatellite in a given infection) and the number of $\left|\boldsymbol{y}_m^{(t)}\right| > 1$ (i.e. the number of heteroallelic microsatellite calls). To see why this is the case, first let $\boldsymbol{H}^{(t)}$ be a matrix whose column vectors are haploid genotypes compatible with $\boldsymbol{y}^{(t)}$ (e.g. Equation (5)).

Example haploid genotypes compatible with $\boldsymbol{y}^{(0)}$ of Equation (2):

$$\boldsymbol{H}^{(0)} = \begin{matrix} m=1 \\ m=2 \\ m=3 \end{matrix} \begin{pmatrix} 2 & 8 & 2 & 8 \\ 7 & 4 & 4 & 7 \\ 3 & 3 & 3 & 3 \end{pmatrix}. \tag{5}$$

For a given $\boldsymbol{y}^{(t)}$, the number of possible haploid genotypes (i.e. number of columns of $\boldsymbol{H}^{(t)}$) is $\prod_{m=1}^{M} \left|\boldsymbol{y}_m^{(t)}\right|$. The number of ways to label vertices in $\boldsymbol{V}^{(t)}$ is given by the number of ways to choose $c^{(t)}$ haploid genotypes from the $\prod_{m=1}^{M} \left|\boldsymbol{y}_m^{(t)}\right|$ possible haploid genotypes,

$$\frac{\prod_{m=1}^{M} \left|\boldsymbol{y}_m^{(t)}\right|!}{\left(\prod_{m=1}^{M} \left|\boldsymbol{y}_m^{(t)}\right| - c^{(t)}\right)!\; c^{(t)}!}. \tag{6}$$

However, of the many combinations given by Equation (6),

$$\mathbb{P}\left(\boldsymbol{y} \mid \boldsymbol{G}_{ab}\right) > 0 \text{ iff } \left(\prod_{t=0}^{T} \prod_{m=1}^{M} \prod_{y_m^{(t)} \in \boldsymbol{y}_m^{(t)}} \mathbb{I}_{\left\{h_{ab_{i_m}}\right\}_{i \in \mathcal{I}^{(t)}}} \left(y_m^{(t)}\right) \right) = 1. \tag{7}$$

That is, only combinations where all alleles in $\boldsymbol{y}$ are represented at least once (e.g. those in Supplementary Fig. 10) lead to $\mathbb{P}\left(\boldsymbol{y} \mid \boldsymbol{G}_{ab}\right) > 0$, and contribute to the total number of ways, A, to label a graph with vertex haploid genotype labels compatible with $\boldsymbol{y}$. Since A is enumerated independently of edge relatedness labels, graphs that have $\mathbb{P}\left(\boldsymbol{y} \mid \boldsymbol{G}_{ab}\right) = 0$ due to $\mathbb{I}_{\text{cln}}\left(k_{ab_{ij}}\right) = 1$ and $\mathbb{I}_{h_{ab_{i_m}}}\left(h_{ab_{j_m}}\right) = 0$ (see below) or NA values do contribute to A.

*

Step two considers the probability of the data given a graph. The probability of the data given a vertex and edge labelled graph, $\mathbb{P}\left(\boldsymbol{y} \mid \boldsymbol{G}_{ab}\right)$, is calculated assuming conditional independence between microsatellites and between edges,

$$\mathbb{P}\left(\boldsymbol{y} \mid \boldsymbol{G}_{\boldsymbol{ab}}\right) = \left\{ \prod_{i=2}^{|G_{ab}|} \prod_{j=1}^{i-1} \prod_{m=1}^{M} \mathbb{P}\left(y_{i_m}, y_{j_m} \mid h_{ab_{i_m}}, h_{ab_{j_m}}, k_{ab_{ij}}\right) \right\}. \tag{8}$$

where $y_{i_m} = \left\{ y_m^{(t)} \in \boldsymbol{y}_m^{(t)} \;:\; y_m^{(t)} = h_{ab_{i_m}} \text{ and } i \in \mathcal{I}^{(t)} \right\}$. Note that y_{i_m} and y_{j_m} may be within or across infections (i.e. i and j may be from within the same or across different $\mathcal{I}^{(0)}, \ldots, \mathcal{I}^{(T)}$).

Hereafter we consider a single graph with fixed vertex and edge labels thus drop the indices a and b. To evaluate $\mathbb{P}\left(y_{i_m}, y_{j_m} \mid h_{i_m}, h_{j_m}, k_{ij}\right)$ we assume conditional independence between pairs of vertices and edges given the IBD state of the mth marker,

$$\mathbb{P}\left(y_{i_m}, y_{j_m} \mid h_{i_m}, h_{j_m}, k_{ij}\right) = \sum_{\text{IBD}=0}^{1} \mathbb{P}\left(y_{i_m}, y_{j_m} \mid h_{i_m}, h_{j_m}, \text{IBD}\right) \mathbb{P}\left(\text{IBD} \mid k_{ij}\right) \tag{9}$$

where

- $\mathbb{P}(\text{IBD} = 1 \mid k_{ij} = \text{cln}) = 1$; $\mathbb{P}(\text{IBD} = 1 \mid k_{ij} = \text{sib}) = 0.5 + \alpha$; $\mathbb{P}(\text{IBD} = 1 \mid k_{ij} = \text{str}) = 0 + \alpha$;
- $\mathbb{P}(\text{IBD} = 0 \mid k_{ij}) = 1 - \mathbb{P}(\text{IBD} = 1 \mid k_{ij})$;
- α is an additive effect of background population-level inbreeding;
- $\mathbb{P}(y_{i_m}, y_{j_m} \mid h_{i_m}, h_{j_m}, \text{IBD} = 1) = 1/2\left(f_{h_{i_m}} + f_{h_{j_m}}\right) = f_{h_{i_m}}$ if $h_{i_m} = h_{j_m}$ and 0 otherwise;
- $\mathbb{P}(y_{i_m}, y_{j_m} \mid h_{i_m}, h_{j_m}, \text{IBD} = 0) = f_{h_{i_m}} \times f_{h_{j_m}}$;
- $f_{h_{i_m}}$ and $f_{h_{j_m}}$ denote the frequency of the allele at the mth microsatellite of the haploid genotype label of the ith and j^{th} vertex, respectively. Equivalently, we could write $f_{y_{i_m}}$ and $f_{y_{i_m}}$, respectively.

Together, the above lead to

$$\begin{aligned}\mathbb{P}(y_{i_m}, y_{j_m} \mid h_{i_m}, h_{j_m}, k_{ij} = \text{str}) &= \left(\alpha f_{h_{i_m}} + (1-\alpha) f_{h_{i_m}} f_{h_{j_m}}\right)^{\mathbb{I}_{h_{i_m}}(h_{j_m})} \times \\ &\quad \left(\alpha \times 0 + (1-\alpha) f_{h_{i_m}} f_{h_{j_m}}\right)^{1-\mathbb{I}_{h_{i_m}}(h_{j_m})}, \quad (10)\\ \mathbb{P}(y_{i_m}, y_{j_m} \mid h_{i_m}, h_{j_m}, k_{ij} = \text{sib}) &= \left((0.5+\alpha) f_{h_{i_m}} + (0.5-\alpha) f_{h_{i_m}} f_{h_{j_m}}\right)^{\mathbb{I}_{h_{i_m}}(h_{j_m})} \times \\ &\quad \left((0.5+\alpha) \times 0 + (0.5-\alpha) f_{h_{i_m}} f_{h_{j_m}}\right)^{1-\mathbb{I}_{h_{i_m}}(h_{j_m})}, \quad (11)\\ \mathbb{P}(y_{i_m}, y_{j_m} \mid h_{i_m}, h_{j_m}, k_{ij} = \text{clo}) &= \left(1 \times f_{h_{i_m}} + 0 \times f_{h_{i_m}} f_{h_{j_m}}\right)^{\mathbb{I}_{h_{i_m}}(h_{j_m})} \times \\ &\quad \left(1 \times 0 + 0 \times f_{h_{i_m}} f_{h_{j_m}}\right)^{1-\mathbb{I}_{h_{i_m}}(h_{j_m})}. \quad (12)\end{aligned}$$

*

Step three considers the probability of a graph given a series of recurrence states. To calculate the probability of a graph given a series of recurrence states, we assume independence between recurrence states,

$$\mathbb{P}(\boldsymbol{G} \mid \boldsymbol{R}) = \prod_{t=1}^{T} \mathbb{P}\left(\boldsymbol{G} \mid R^{(t)}\right) \quad (13)$$

where

$$\mathbb{P}\left(\boldsymbol{G} \mid R^{(t)} = L\right) = \frac{1}{AB} \quad (14)$$

$$\mathbb{P}\left(\boldsymbol{G} \mid R^{(t)} = I\right) = \begin{cases} \frac{1}{AB_I} & \text{if } \forall z < t, \forall i \in \mathcal{I}^{(t)}, \forall j \in \mathcal{I}^{(z)}\ k_{ij} = \text{str} \\ 0 & \text{otherwise,} \end{cases} \quad (15)$$

$$\mathbb{P}\left(\boldsymbol{G} \mid R^{(t)} = C\right) = \begin{cases} \frac{1}{AB_C} & \text{if } \exists\ c^{(t)} \text{disjoint pairs } ij : i \in \mathcal{I}^{(t)} \text{ and } j \in \mathcal{I}^{(t-1)} \text{ and } k_{ij} = \text{cln}, \\ 0 & \text{otherwise,} \end{cases} \quad (16)$$

where $B_I < B$ and $B_C < B$ denote the number of graphs that satisfy the conditions outlined in Equation (15) and (16), both of which are determined algorithmically from the adjacency matrix of $\boldsymbol{G}$.

The condition outlined in Equation (16) that $j \in \mathcal{I}^{(t-1)}$ specifies that a recrudescence is seeded by the most recent past infection only (assumption 2). Also in Equation (16), the condition that there are $c^{(t)}$ disjoint pairs follows from assumptions 5 and 6 and results in zero probability of recrudescence following an infection with lower COI (i.e. a recrudescence cannot be more diverse that the infection that seeded it - diversity cannot be be created, only lost). For example, in Equation (2), $R_n^{(2)}$ has zero probability of being a recrudescence because $c^{(2)} = 2 > c^{(1)} = 1$.

Presently, Equation (14) to (16) do not take into the relative likelihood of parasites that are strangers, siblings or clones in relation to one another within an inoculation. Doing so is theoretically possible (e.g. by coupling to a transmission model), but very difficult without prior knowledge of the relative proportions expected given different recurrence states. A full understanding requires joint modelling of the hidden recurrence states, transmission (to capture the expected counts of strangers and siblings in co-inoculations, thus treating COI as a random variable) and of the impact of host covariates (e.g. age, treatment history, etc.) on the hypnozoite bank to better understand the observed variance in genetically homologous versus heterologous relapse [9,13–19]. Note that $\mathbb{P}(\boldsymbol{G} \mid \boldsymbol{R})$ implicitly conditions on $\boldsymbol{y}$ via $c^{(t)}$.

Implementation of the genetic model: Above, $t = 0, \dots, T_n$ where T_n is the number of recurrences experienced by the nth individual. In the code, $t = 1, \dots, T_n$ where T_n is the number of infections experienced by the nth individual.

The model is implemented on the log scale to prevent under and over flow problems, using the 'log-sum-exp' trick where appropriate. Instead of summing over a and b in one step as Equation (4) implies, we first sum over graphs indexed by $a = 1, \dots, A$ fixing b and working entirely on the log domain (interior of square brackets Equation (17)); we then sum over graphs indexed by $b = 1, \dots, B_{R^{(t)}}$ where $B_{R^{(t)}} \in \{B_L = B, B_C, B_I\}$ is determined algorithmically from the adjacency matrix of $\boldsymbol{G}$,

$$
\begin{aligned}
\log \mathbb{P}(\boldsymbol{y} \mid \boldsymbol{R}) &= \log \left(\sum_{b=1}^{B} \sum_{a=1}^{A} \mathbb{P}(\boldsymbol{y} \mid \boldsymbol{G}_{ab}) \, \mathbb{P}(\boldsymbol{G}_{ab} \mid \boldsymbol{R}) \right), \\
&= \log \left(\sum_{b=1}^{B_{R^{(t)}}} \sum_{a=1}^{A} \mathbb{P}(\boldsymbol{y} \mid \boldsymbol{G}_{ab}) \, \frac{1}{A \times B_{R^{(t)}}} \right), \\
&= \log \left(\sum_{b=1}^{B_{R^{(t)}}} \sum_{a=1}^{A} \mathbb{P}(\boldsymbol{y} \mid \boldsymbol{G}_{ab}) \, \frac{1}{A} \right) - \log B_{R^{(t)}}, \\
&= \log \left(\sum_{b=1}^{B_{R^{(t)}}} \exp \left[\log \left(\sum_{a=1}^{A} \exp \left(\log \mathbb{P}(\boldsymbol{y} \mid \boldsymbol{G}_{ab}) \right) \right) - \log A \right] \right) - \log B_{R^{(t)}}, \qquad (17)
\end{aligned}
$$

where the subscript n is dropped since n is fixed.

When $\boldsymbol{H}^{(t)}$ is large it is very difficult to deterministically enumerate all $a \in \{1, \dots, A\}$[1], since the number of combinations counted by Equation (6) is huge, even if only a small number are compatible with the observed data according to Equation (7). As such, when the number of haploid genotypes in $\boldsymbol{H}^{(t)}$ exceeds a threshold (currently set to 50), we permute and bootstrap the observed data to create combinations that are guaranteed compatible with the data[2]. Specifically, we permute saturated heteroallelic markers (markers where $\mid \boldsymbol{y}_{n_m}^{(t)} \mid = c_n^{(t)}$), and bootstrap heteroallelic markers with redundancy (markers where $\mid \boldsymbol{y}_{n_m}^{(t)} \mid < c_n^{(t)}$). Since this is a probablistic approach some of the combinations are duplicated; duplicate combinations are subsequently removed. Rather than generating a fixed number of combinations, we generate combinations until either the number of unique combinations stabilises over three consecutive iterations or exceeds a threshold, currently set to 500 (a threshold ensures finite compute time for highly complex infections). If stability is met, we almost certainly recover all $a \in \{1, \dots, A\}$; otherwise, we return a notification that notifies that probabilistic phasing did not stabilise.

[1] To deterministically enumerate all $a \in \{1, \dots, A\}$, we first enumerate combinations counted by Equation (6) using the combinations function from the R package gtools[20]. We then discard all those that are incompatible with the observed data according to Equation (7). This approach guarantees summation over all $a \in \{1, \dots, A\}$ (Equation (4)), but is inefficient since many combinations are incompatible according to Equation (7).

[2] A more efficient deterministic solution, which is beyond the scope of the current study, would use dynamic programming. It would remove the need for a threshold over the number of haploid genotypes in $\boldsymbol{H}^{(t)}$, but not the need for the threshold over the number of compatible combinations, since the number of compatible combinations is also used to avoid computationally prohibitive computations of Equation (4).

Viable graph brute-force search algorithm: For a given set of complexities $c_n^{(0)}, \ldots, c_n^{(T_n)}$ we implement a brute-force algorithm that searches over all graphs and stores all viable graphs. Viable graphs can be described with two independent rules:

(a) No clonal edges between vertices within an infection

(b) All connected paths must obey the transitivity property

The algorithm, described here for $\alpha = 0$, is summarised as follows:

1. Construct a list $\mathcal{S}_G$ of all graphs by listing all adjacency matrices described by block matrices (one per infection) for which the block matrices only contain $\{0, 0.5\}$, and the across blocks contain $\{0, 0.5, 1\}$ (this implies no clonal edges within infections).

2. For each $G \in \mathcal{S}_G$:

   - Enumerate all connected components in G and verify that each connected component is a clique (fully connected subgraph).
   - List all triangular cliques (fully connected subgraphs containing exactly three vertices)
   - For each triangular clique G' compute the sum of the weighted edges: If the sum of the edges in G' is equal to 2.5 then Reject G else Accept the subgraph G'

3. If all subgraphs are accepted, Accept G

Step 2 results in obeying rule (b) whereby graphs with non-transitive relatedness patterns (e.g. A is clonal with both B & C but B is sibling with C) are rejected.

Joint time-to-event and genetic model: Time-to-event data provide intermediate level evidence for or against relapse and ignore rich signals from genetic data. Alone, genetic data do not suffice to pinpoint relapsing infections as unrelated parasites are found both within and across inocula, and are compatible with relapse or reinfection. However, by combining both sources of information we can use genetic data to update the *a priori* belief of recurrent states based on the time-to-event. In this work we combine the two models informally, using the posterior of the time-to-event model as a discrete prior in the genetic model and assuming conditional independence of time-to-event and genetic information as described below. It remains to be seen whether a formal joint model of both data types would add value[21].

Conditional independence of time-to-event and genetic information: For the nth individual, let $\boldsymbol{x}_n$ denote all available time-to-event data, $\boldsymbol{y}_n$ denote all available genetic data, and $\boldsymbol{R}_n$ denote hidden recurrent states. Under the joint model of relapse we assume $\boldsymbol{x}_n$ and $\boldsymbol{y}_n$ are conditionally independent given $\boldsymbol{R}_n$:

$$\mathbb{P}\left(\boldsymbol{x}_n, \boldsymbol{y}_n | \boldsymbol{R}_n\right) = \mathbb{P}\left(\boldsymbol{x}_n | \boldsymbol{R}_n\right) \mathbb{P}\left(\boldsymbol{y}_n | \boldsymbol{R}_n\right) \tag{18}$$

This assumption allows sequential update of the posterior probability of $\boldsymbol{R}_n$. First given information from the time-of-event data,

$$\mathbb{P}\left(\boldsymbol{R}_n | \boldsymbol{x}_n\right) = \frac{\mathbb{P}\left(\boldsymbol{x}_n | \boldsymbol{R}_n\right) \mathbb{P}\left(\boldsymbol{R}_n\right)}{\mathbb{P}\left(\boldsymbol{x}_n\right)}; \tag{19}$$

second given information from genotyping (be it microsatellite data or other),

$$\mathbb{P}\left(\boldsymbol{R}_n | \boldsymbol{y}_n, \boldsymbol{x}_n\right) = \frac{\mathbb{P}\left(\boldsymbol{y}_n | \boldsymbol{R}_n\right) \mathbb{P}\left(\boldsymbol{R}_n | \boldsymbol{x}_n\right)}{\sum_{\text{All}\boldsymbol{R}_n} \mathbb{P}\left(\boldsymbol{y}_n | \boldsymbol{R}_n\right) \mathbb{P}\left(\boldsymbol{R}_n | \boldsymbol{x}_n\right)}. \tag{20}$$

The conditional independence assumption can be interpreted as no propensity for relapsing stranger parasites to occur at different time intervals from relapsing parasites that are related. This assumption does not hold under the current model of microscopically-detectable recurrence if immunity following a symptomatic (patent) infection develops such that antigenically related blood-stage parasites, which are the asexual progeny of genetically related hypnozoites, are systematically more likely to be suppressed thereby cause sub-microscopic relapses that are not detected. Acquisition of strain-specific blood-stage *P. vivax* immunity was observed in malaria therapy and challenge studies[1] with suppression of relapses[22]; and there is comparable evidence of strain-specific blood-stage *P. falciparum* immunity in endemic populations[23]. The quantitative determinants of strain-specific immunity to *P. vivax* in endemic populations, and the antigenicity and thus immune responses to strains that are related but not clonal merits further research. Sub-microscopic considerations aside, White[1] hypothesises that malarial illness itself activates pre-existent hypnozoites which in long-latency *P. vivax* could lead to a preferential activation of genetically unrelated hypnozoites[1]. In long-latency vivax, reinfection would thereby trigger activation of previously inoculated parasites (unrelated) and the most recently accumulated hypnozoites would stay dormant for 8-9 months. This is highly speculative, however.

Propagation of uncertainty: We use Monte Carlo sampling to numerically approximate $\mathbb{P}\left(\boldsymbol{R}_n | \boldsymbol{x}_n\right)$, Equation 19. We then draw from the numerical approximation uniformly at random (while also drawing from the posterior allele frequency distributions) to recover a numerical approximation of $\mathbb{P}\left(\boldsymbol{R}_n | \boldsymbol{x}_n, \boldsymbol{y}_n\right)$. Since we explicitly sum over all R_n, for a given allele frequency draw Equation (20) can be considered a transformation of the sample approximating $\mathbb{P}\left(\boldsymbol{R}_n | \boldsymbol{x}_n\right)$. Uncertainty due

to limited genetic data is not propagated through this transformation (it is computationally prohibitive to do so at present: each transformation would require the parametric bootstrap thus full simulation of all graphs under the genetic model). Nevertheless, the Bayesian transformation guards against over interpretation of limited data: when data are few the genetic model is conservative and returns estimates close to the prior.

Supplementary Note

Relatedness between meiotic siblings: The average pairwise relatedness between sporozoites that are meiotic siblings is 0.5, despite average pairwise relatedness between haploid meiotic products (hereafter referred to as HMPs) being 0.33 [7,24]. This is because of the massive expansion of the HMPs during sporogeny [4,5]. In short, expansion amounts to there being $\frac{8}{16} = 0.5$ IBD permutations of sporozoites from the mature oocyst, despite $\frac{2}{6} = 0.33$ IBD combinations of HMPs from the tetraploid zygote [7,24]. A more detailed explanation follows.

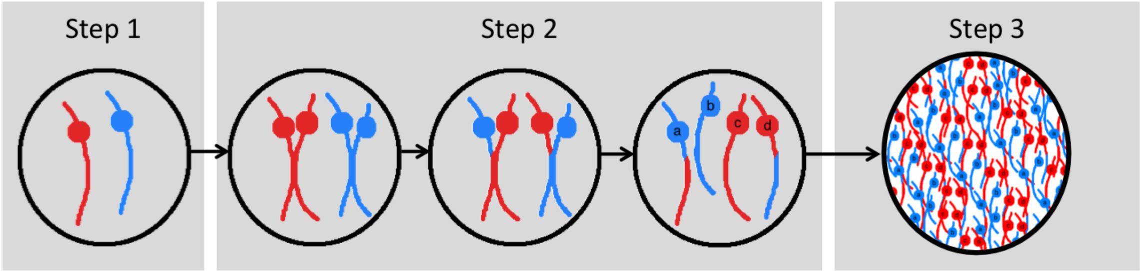

Supplementary Fig. 11: A schematic of sexual recombination between genetically distinct malaria parasites based on a review [5 5]. Different colours denote different genomes. A single locus is highlighted by a solid circle. Two haploid gametes (one micro, one macro) fuse forming a diploid zygote (step 1). Endomeiotic replication follows, resulting in a tetraploid zygote with four presumed HMPs, labelled *a, b, c* and *d* (step 2). Note that the labels a to c refer to the HMPs in their entirety, not the alleles at the highlighted locus. The zygote then transforms into a motile ookinete, where the second round of meiosis is thought to occur, before maturation of the ookinete into an oocyst. Within the oocyst, the four presumed haploid meiotic products replicate by endomitosis producing thousands of genomes that mature into sporozoites (step 3).

Supplementary Fig. 11 is a schematic of sexual recombination between genetically distinct malaria parasites based on a review [5]. Different colours denote different genomes. The microgamete is blue, and the macrogamete is red. A single locus is highlighted by a solid circle. In step 1, two genetically distinct haploid gametes (one micro, one macro, represented by blue and red, respectively) come together forming a diploid zygote. Endomeiotic replication follows, resulting in a tetraploid zygote with four presumed HMPs (step 2). The zygote then transforms into a motile ookinete before maturation into an oocyst, where, in a process called sporogony, the four presumed haploid meiotic products replicate by endomitosis producing thousands of genomes that mature into sporozoites (step 3).

Since there are only four HMPs in the tetraploid zygote (a, b, c and d in Supplementary Fig. 11), there are only $\binom{4}{2} = 6$ ways to select distinct pairs of HMPs (ab, ac, ad, bc, bd and cd). A pair is identical by descent (IBD=1) at a given locus only if the HMPs share a marker inherited from the same gamete (otherwise IBD=0). At any given locus, 2 HMPs inherit markers from the microgamete (a and b at the highlighted locus in Supplementary Fig. 11), there is thus $\binom{2}{2} = 1$ way of choosing two markers derived from the microgametes (ab in this example). Similarly, there

is $\binom{2}{2} = 1$ way of selecting two markers derived from the macrogamete (cd in this example). Of the 6 HMP pairs, there are thus two that are identical by descent (IBD=1: ab and cd) and 4 that are not (IBD=0: ac, ad, bc, bd) amounting to an average pairwise relatedness between HMPs of $\frac{2}{6} = 0.33$.

Since within an oocyst there are thousands of sporozoites derived from only 4 HMPs we can select sporozoites derived from the same and different HMPs (i.e. equivalent to sampling HMPs with replacement). Given the four HMP precursors, there are in total $4 \times 4 = 16$ ways to select sporozoites (*aa, ab, ac, ad, ba, bb, bc, bd, ca, cb, cc, cd, da, db, dc, dd*). Given two of the four HMP precursors inherit markers from the microgamete (a and b), there are $2 \times 2 = 4$ ways to select two sporozoites that have markers derived from the microgametes (*aa, bb, ab, ba*). Similarly there are $2 \times 2 = 4$ ways to select two markers derived from the macrogamete (*cc, dd, cd, dc*). Of the 16 sporozoite pairwise comparisons, $4 + 4 = 8$ are thus IBD amounting to an average pairwise relatedness between sporozoites of $8/16 = 0.5$.

If we condition on sporozoites being different at least at one locus (e.g. excluding repeats *aa, bb, cc, dd* from the numerator and denominator), the average pairwise relatedness between remaining sporozoites is $4/12 = 0.33$. This means that the expected relatedness between two genetically distinct sporozoites that are meiotic siblings is 0.33. On the opposite end of the spectrum, if genetically identical micro and macro gametes self-fertilize all combinations and permutations are IBD and the haploid meiotic products are clonal.

Supplementary References

[1] White, N. J. Determinants of relapse periodicity in Plasmodium vivax malaria. *Malaria Journal* **10**, 297 (2011).

[2] Schmidt, L. Compatibility of relapse patterns of plasmodium cynomolgi infections in rhesus monkeys with continuous cyclical development and hypnozoite concepts of relapse. *The American Journal of Tropical Medicine and Hygiene* **35**, 1077–1099 (1986).

[3] Hoffman, J. I. & Amos, W. Microsatellite genotyping errors: Detection approaches, common sources and consequences for paternal exclusion. *Molecular Ecology* **14**, 599–612 (2005).

[4] Matthews, H., Duffy, C. W. & Merrick, C. J. Checks and balances? DNA replication and the cell cycle in Plasmodium. *Parasites & vectors* **11**, 216 (2018).

[5] Baton, L. A. & Ranford-Cartwright, L. C. Spreading the seeds of million-murdering death: metamorphoses of malaria in the mosquito. *Trends in Parasitology* **21**, 573–580 (2005).

[6] Bink, M. C., Anderson, A. D., Van De Weg, W. E. & Thompson, E. A. Comparison of marker-based pairwise relatedness estimators on a pedigreed plant population. *Theoretical and Applied Genetics* **117**, 843–855 (2008).

[7] Zhu, S. J. *et al.* The origins and relatedness structure of mixed infections vary with local prevalence of P. falciparum malaria. *eLife* **8**, e40845 (2019).

[8] Soontarawirat, I. *et al.* Plasmodium vivax genetic diversity and heterozygosity in blood samples and resulting oocysts at the Thai-Myanmar border. *Malaria Journal* **16**, 355 (2017).

[9] Imwong, M. *et al.* Relapses of Plasmodium vivax infection usually result from activation of heterologous hypnozoites. *The Journal of Infectious Diseases* **195**, 927–33 (2007).

[10] Gunawardena, S. *et al.* Geographic structure of Plasmodium vivax: microsatellite analysis of parasite populations from Sri Lanka, Myanmar, and Ethiopia. *The American Journal of Tropical Medicine and Hygiene* **82**, 235–242 (2010).

[11] Schaffner, S. F., Taylor, A. R., Wong, W., Wirth, D. F. & Neafsey, D. E. HmmIBD: Software to infer pairwise identity by descent between haploid genotypes. *Malaria Journal* **17**, 10–13 (2018).

[12] Henden, L., Lee, S., Mueller, I., Barry, A. & Bahlo, M. Identity-by-descent analyses for measuring population dynamics and selection in recombining pathogens. *PLoS Genetics* **14**, 1–31 (2018).

[13] Orjuela-Sanchez, P., da Silva, N. S., da Silva-Nunes, M. & Ferreira, M. U. Recurrent parasitemias and population dynamics of Plasmodium vivax polymorphisms in rural Amazonia. *American Journal of Tropical Medicine and Hygiene* **81**, 961–968 (2009).

[14] Veron, V. *et al.* Genetic diversity of msp3alpha and msp1b5 markers of Plasmodium vivax in French Guiana. *Malaria Journal* **8**, 40 (2009).

[15] Restrepo, E., Imwong, M., Rojas, W., Carmona-Fonseca, J. & Maestre, A. High genetic polymorphism of relapsing P. vivax isolates in northwest Colombia. *Acta Tropica* **119**, 23–29 (2011).

[16] de Araujo, F. C., de Rezende, A. M., Fontes, C. J., Carvalho, L. H. & Alves de Brito, C. F. Multiple-clone activation of hypnozoites is the leading cause of relapse in Plasmodium vivax infection. *PLoS ONE* **7**, e49871 (2012).

[17] Lin, J. T. *et al.* Plasmodium vivax isolates from Cambodia and Thailand show high genetic complexity and distinct patterns of P. vivax multidrug resistance gene 1 (pvmdr1) polymorphisms. *American Journal of Tropical Medicine and Hygiene* **88**, 1116–1123 (2013).

[18] Maneerattanasak, S. *et al.* Molecular and immunological analyses of confirmed Plasmodium vivax relapse episodes. *Malaria Journal* **16**, 228 (2017).

[19] Popovici, J. *et al.* Genomic analyses reveal the common occurrence and complexity of plasmodium vivax relapses in cambodia. *mBio* **9**, e01888–17 (2018).

[20] Warnes, G. R., Bolker, B. & Lumley, T. *gtools: Various R Programming Tools* (2018). URL `https://CRAN.R-project.org/package=gtools`. R package version 3.8.1.

[21] Jacob, P. E., Murray, L. M., Holmes, C. C. & Robert, C. P. Better together? statistical learning in models made of modules. *Preprint at https://arxiv.org/abs/1708.08719* (2017).

[22] Boyd, M. F. *et al.* A review of studies on immunity to vivax malaria. *Journal of the National Malaria Society* **6**, 12–31 (1947).

[23] Early, A. M. *et al.* Host-mediated selection impacts the diversity of plasmodium falciparum antigens within infections. *Nature Communications* **9**, 1381 (2018).

[24] Wong, W., Wenger, E. A., Hartl, D. L. & Wirth, D. F. Modeling the genetic relatedness of Plasmodium falciparum parasites following meiotic recombination and cotransmission. *PLOS Computational Biology* **14**, e1005923 (2018).
