# Supplementary material for: Resolving the cause of recurrent Plasmodium vivax malaria probabilistically
Source: Nat Commun. 2019 Dec 6;10:5595. doi: 10.1038/s41467-019-13412-x (PMC6898227; doi:10.1038/s41467-019-13412-x)
Supplement: Supplementary file 3 — Reporting Summary [file 41467_2019_13412_MOESM3_ESM.pdf]

nature research

Corresponding author(s): Aimee R Taylor, James A Watson, Nicholas J White

Last updated by author(s): Oct 11, 2019

Reporting Summary

Nature Research wishes to improve the reproducibility of the work that we publish. This form provides structure for consistency and transparency in reporting. For further information on Nature Research policies, see Authors & Referees and the Editorial Policy Checklist.

Statistics

For all statistical analyses, confirm that the following items are present in the figure legend, table legend, main text, or Methods section.

n/a	Confirmed	
☐	☒	The exact sample size (*n*) for each experimental group/condition, given as a discrete number and unit of measurement
☐	☒	A statement on whether measurements were taken from distinct samples or whether the same sample was measured repeatedly
☒	☐	The statistical test(s) used AND whether they are one- or two-sided<br>*Only common tests should be described solely by name; describe more complex techniques in the Methods section.*
☐	☒	A description of all covariates tested
☐	☒	A description of any assumptions or corrections, such as tests of normality and adjustment for multiple comparisons
☐	☒	A full description of the statistical parameters including central tendency (e.g. means) or other basic estimates (e.g. regression coefficient) AND variation (e.g. standard deviation) or associated estimates of uncertainty (e.g. confidence intervals)
☐	☒	For null hypothesis testing, the test statistic (e.g. *F*, *t*, *r*) with confidence intervals, effect sizes, degrees of freedom and *P* value noted<br>*Give P values as exact values whenever suitable.*
☐	☒	For Bayesian analysis, information on the choice of priors and Markov chain Monte Carlo settings
☐	☒	For hierarchical and complex designs, identification of the appropriate level for tests and full reporting of outcomes
☒	☐	Estimates of effect sizes (e.g. Cohen's *d*, Pearson's *r*), indicating how they were calculated

Our web collection on statistics for biologists contains articles on many of the points above.

Software and code

Policy information about availability of computer code

Data collection	No software was used for the data collection process
Data analysis	All software used was tailor made to this particular problem and the reported model is central to the results of the paper. The software was written using R (versions 3.4.3 (JAW) and 3.5.0 (ART)) and using rstan (version 2.18.1) for Markov Chain Monte Carlo estimation. All software is publicly available in a github repository which can be found at: https://github.com/jwatowatson/RecurrentVivax (DOI: 10.5281/zenodo.3368828)

For manuscripts utilizing custom algorithms or software that are central to the research but not yet described in published literature, software must be made available to editors/reviewers. We strongly encourage code deposition in a community repository (e.g. GitHub). See the Nature Research guidelines for submitting code & software for further information.

Data

Policy information about availability of data

All manuscripts must include a data availability statement. This statement should provide the following information, where applicable:

- Accession codes, unique identifiers, or web links for publicly available datasets
- A list of figures that have associated raw data
- A description of any restrictions on data availability

Data used are open access and are in the form of .RData files in the github repository (DOI: 10.5281/zenodo.3368828)

Field-specific reporting

Please select the one below that is the best fit for your research. If you are not sure, read the appropriate sections before making your selection.

☒ Life sciences ☐ Behavioural & social sciences ☐ Ecological, evolutionary & environmental sciences

For a reference copy of the document with all sections, see nature.com/documents/nr-reporting-summary-flat.pdf

Life sciences study design

All studies must disclose on these points even when the disclosure is negative.

Sample size	No sample size calculations applied here. We analysed available genetic and epidemiological data from two large clinical trials in P. vivax
Data exclusions	Some recurrent vivax infections were excluded from the genetic model due to computational complexity, this is noted in the Methods. No recurrences were excluded from the time-to-event model.
Replication	No replication attempts have been done yet. We are in the process of generating whole genome sequencing for some samples for replication in future work
Randomization	Both clinical trials had randomised drug allocation. In the BPD trial all patients received primaquine but randomised to 7 or 14 days regimens. In VHX, patients were randomised to primaquine plus chloroquine, chloroquine monotherapy, or 7 days artesunate monotherapy. In our analysis we combined data from all primaquine and non-primaquine treated patients
Blinding	Drug treatment was unblinded for both trials

Reporting for specific materials, systems and methods

We require information from authors about some types of materials, experimental systems and methods used in many studies. Here, indicate whether each material, system or method listed is relevant to your study. If you are not sure if a list item applies to your research, read the appropriate section before selecting a response.

Materials & experimental systems

n/a	Involved in the study	
☒	☐	Antibodies
☒	☐	Eukaryotic cell lines
☒	☐	Palaeontology
☒	☐	Animals and other organisms
☐	☒	Human research participants
☐	☒	Clinical data

Methods

n/a	Involved in the study	
☒	☐	ChIP-seq
☒	☐	Flow cytometry
☒	☐	MRI-based neuroimaging

Human research participants

Policy information about studies involving human research participants

Population characteristics	The patient population comprised migrant workers and displaced persons of Burman and Karen ethnicities living along the Thailand-Myanmar border presenting to SMRU outpatient clinics.
Recruitment	All patients ≥6 months and weighing ≥7 kg presenting to SMRU outpatient clinics with microscopy-confirmed uncomplicated P. vivax monoinfections were screened. Exclusion criteria included pregnancy, severe malaria, hematocrit <25%, allergies to antimalarial drugs, blood transfusion in the last 3 months, antimalarial use in the last 4 weeks, and inability to comply with study procedures.<br>In the BPD study, G6PD deficiency by fluorescent spot test was also an exclusion criteria.
Ethics oversight	The BPD study was approved by both the Mahidol University Faculty of Tropical Medicine Ethics Committee (MUTM 2011–043, TMEC 11-008) and the Oxford Tropical Research Ethics Committee (OXTREC 17-11). The VHX study was approved by the Mahidol University Faculty of Tropical Medicine Ethics Committee (MUTM 2010-006) and the Oxford Tropical Research Ethics Committee (OXTREC 04-10).

Note that full information on the approval of the study protocol must also be provided in the manuscript.

Clinical data

Policy information about clinical studies

All manuscripts should comply with the ICMJE guidelines for publication of clinical research and a completed CONSORT checklist must be included with all submissions.

Clinical trial registration	NCT01074905 and NCT01640574
Study protocol	The full trial protocols can be accessed by contacting the principal investigator of both studies: Professor Francois Nosten francois@tropmedres.ac
Data collection	The first study (VHX) ran from 2010 to 2012. The second study (BPD) ran from 2012 to 2014. Both studies were done in the clinics operated by the Shoklo Malaria Research Unit along the Thailand-Myanmar border.
Outcomes	Recurrence with Plasmodium vivax malaria within 52 weeks of first treatment dose
